# Factors of success, barriers, and the role of frontline workers in Indigenous maternal-child health programs: a scoping review

**DOI:** 10.1186/s12939-024-02118-2

**Published:** 2024-02-13

**Authors:** Charlene Thompson, Tara Million, Devan Tchir, Angela Bowen, Michael Szafron

**Affiliations:** 1 College of Nursing, University of Saskatchewan, Health Sciences Building – 1A10, 107 Wiggins Road, Box 6, Saskatoon, SK S7N 5E5 Canada; 2 Department of Indigenous Studies, University of Lethbridge, A410 University Hall, Lethbridge, AB T1K 3M4 Canada; 3 Alberta Health Services, Edmonton, AB Canada; 4 School of Public Health, University of Saskatchewan, 104 Clinic Place, Saskatoon, SK S7N 2Z4 Canada

**Keywords:** Indigenous peoples, Maternal-child health, Health program planning and evaluation, Frontline workers, Scoping review

## Abstract

**Background:**

Despite considerable investment in maternal-child programs in Canada, there has been little positive impact on the health of Indigenous mothers and their children. The reasons for this are unclear and there is a need to identify how such programs can be successfully implemented. Community input is essential for successful programs; however, it is unclear what the contributions of frontline workers have been in the health program process, i.e., program development, delivery, and evaluation. Based on these identified gaps, this scoping review aimed to: (1) identify factors of success and barriers to successful Indigenous maternal-child community health programs for mothers and their children aged 0–6 years; and (2) explore how frontline workers are included in the program process.

**Methods:**

This scoping review was completed using the Arksey and O’Malley framework, informed by Levac et al. Four data bases (Medline, CINAHL, Embase, and Scopus), grey literature, and reference lists were searched for relevant materials from 1990–2019. Data was extracted from included articles and analysed using descriptive statistics, thematic analysis with the Braun and Clarke framework, and a Principal Component Analysis.

**Results:**

Forty-five peer-reviewed and grey articles were included in the review. Factors of program success included: relationship building; cultural inclusion; knowledge transmission styles; community collaboration; client-centred approaches; Indigenous staff; and operational considerations. Barriers included: impacts of colonization; power structure and governance; client and community barriers to program access; physical and geographical challenges; lack of staff; and operational deficits. Frontline workers were found to have a role in program delivery (*n* = 45) and development (*n* = 25). Few (*n* = 6) had a role in program evaluation.

**Conclusion:**

Although a better understanding of the frontline worker role in maternal-child health programs was obtained from the review, in a large proportion of literature the authors could not determine if the role went beyond program delivery. In addition, no direct input from frontline workers and their perspectives on program success or barriers were identified, suggesting areas to explore in future research. This review's findings have been applied to inform a community-based participatory research project and may also help improve the development, delivery, and evaluation of Indigenous maternal-child health programs.

**Supplementary Information:**

The online version contains supplementary material available at 10.1186/s12939-024-02118-2.

## Background

Health inequity is one of the key challenges to Indigenous maternal-child health [1, 2]. Indigenous mothers and children experience a greater proportion of negative health outcomesand reduced access to care when compared to non-Indigenous mothers and children [2–4]. The increased burden resulting from health inequities negatively impacts mothers' health statuses and is a significant barrier to the growth and development of children [3, 5].

### Indigenous maternal-child health programs

Maternal-child health programs have an essential role in improving the health of Indigenous mothers and children and reducing health inequity [4, 6, 7]. In this context, Indigenous maternal-child health programs are an action or approach in the community setting aimed at mothers and their children to create a positive health impact [4, 6, 8]. Although there are a large number and variety of available maternal-child health programs, there has been little positive impact on the health status of Indigenous mothers and their children [4, 9].Indigenous mothers continue to experience higher rates of gestational diabetes, postpartum diabetes, obesity, anxiety, and depression [10]. Indigenous children experience higher rates of preterm births, sudden infant death syndrome, and higher overall mortality rates [2]. With maternal-child health programs having little effect, there is a need to identify elements that can assist or hinder program success and, potentially, inform current practice [4, 7, 11].

Health programs have been successful when the community is included in the health program process, i.e., the development, delivery, and evaluation of a program [7, 12, 13].. Frontline workers are one aspect of community input that can contribute to program success [4, 14, 15],but it is unclear what their contributions have been in the health program process. Examining the available literature may provide insight into the role of frontline workers in the health program process and ways health program planning and evaluation may be improved. Hence this scoping review aimed to: (1) identify factors of success and barriers to successful Indigenous maternal-child community health programs for Indigenous mothers and their children aged 0–6 years; and (2) explore how frontline workers are included in the Indigenous maternal-child community health program process.

## Methods

### Scoping review rationale

One key challenge in Indigenous health program literature is evaluating research based on a western standard that does not fit the community or community definition of success [16–18]. This challenge has created a body of program evidence that has been criticized as weak [7, 18, 19].) Excluding literature based on quality alone from a review could result in the loss of valuable research that reflects the community and limit the usefulness of the review [16, 20]. A scoping review eliminates a quality assessment from the review process, thus broadening the scope of literature beyond the western standard of evidence and generating relevant results [16, 20, 21] to inform Indigenous maternal-child health programs. Unlike other types of literature reviews, a scoping review is more likely to include a variety of study methods and designs [21]. The scoping review framework is an iterative process, where the team may revisit and refine the stages to ensure comprehensive and pertinent answers to the research questions [20, 21]. Consequently, a scoping review fits the context of a review of literature pertaining to Indigenous maternal-child health where multiple methods, such as randomized control trials, community-based participatory research, and descriptive studies, have been used in health program research [13, 15, 22].

### Scoping review process

This scoping review followed the framework that was developed by Arksey and O’Malley [21] and modified by Levac et al. [20] because Levac et al. [20] enhanced the Arksey and O’Malley framework [21] to include greater guidance to the methodology and build on the consistency of its application in the review process. Six stages make up the framework and include: Stage 1: identifying the research question; Stage 2: identifying relevant studies; Stage 3: study selection; Stage 4: charting the data; Stage 5: collating, summarizing, and reporting the results; and Stage 6: consultation. In an effort to strengthen the rigour of the scoping review, we followed the recommendation of Levac et al. [20] and formed a multi-disciplinary team, CT (team lead), TM, DT, AB, and MS, from public health, nursing, and Indigenous Studies to complete the review.

### Implementation of the process

#### Stage 1: research questions

Based on the aims of our scoping review, the team collaboratively generated two research questions to guide our review:

1. For Indigenous mothers and their children aged 0–6 years, what are the factors of success and barriers to successful Indigenous maternal-child community health programs?
2. How are frontline workers included in the Indigenous maternal-child community health program process?

### Concepts of interest

The team discussed and determined three concepts underlying the research questions needed to be defined: Indigenous Peoples, Indigenous maternal-child community health programs and frontline workers. The team developed the conceptual definitions below using multiple literature sources.

#### Indigenous peoples

In the context of this study, Indigenous Peoples identifies the ‘First Peoples’ or those that inhabited countries such as Canada, Australia, New Zealand, and the United States before colonization [23]. Indigenous Peoples have distinct languages, cultures, and beliefs with strong connections to lands, territories, and resources [24].

#### Indigenous maternal-child community health program

An Indigenous maternal-child community health program was considered to be an action or approach aimed at one or more levels, i.e., the individual, family, whole community, policy, to reduce the mortality rates of women and children and improve their health and well-being [2, 6, 14, 25–27].

#### Frontline workers

Frontline workers are individuals involved in some aspect of the health program process [4, 12, 14, 15]. Examples of frontline workers include nurses, Indigenous Health and Community Workers, midwives, counsellors, peer support workers, and family support workers [3, 13, 28–33].

#### Stage 2: identifying relevant studies

##### Search strategy

The scoping review team consulted with a health sciences librarian to obtain advice on the search parameters and search strategy. We included both peer-reviewed and grey literature in our search. For this review, unless preceded by “peer-reviewed” or “grey”, the terms “articles” and “literature” refer to the combined peer-reviewed and grey literature. Articles were restricted to those written in English.

The literature was limited to Australia, Canada, New Zealand, and the United States based on these countries:

1. Similar histories of colonization [19, 34]
2. Significant populations of Indigenous peoples with similar health status [34–36]
3. scoring near the top of the good health and living standards in the United Nations Development Programme Human Development Index [35]; and
4. the program was implemented in the country.

Articles were narrowed to the timeframe of 1990 to 2019 to capture the developments in health promotion occurring after the introduction of the Ottawa Charter (1986) that defined the components and strategies of health promotion still being applied in current health programs and public health practice [37, 38]. From the Ottawa Charter: health promotion was defined as a process that places the control with people to take a participatory role in improving their health; health is considered a state of physical, social, and mental well-being; health is influenced by external determinants such as education, income, and equity; and health promotion actions were established, such as building health policy, creating supportive environments, and developing personal skills [37, 38].

#### Search terms

Search terms were developed in consultation with a health sciences librarian. The medical subject headings (MeSH) found in search sources, i.e., scholarly databases, and keywords specific to Indigenous Peoples and Indigenous maternal-child health community health programs informed the development of the search term strategy. The following search terms were applied in the search strategy: (Indigenous Peoples of Canada filters [39, 40] OR Oceanic Ancestry Group OR Indigenous OR American Indian OR Indians, North American OR Aboriginal OR Native American) AND (Maternal-Child Health Services OR Child Health OR Child Health Services OR prenatal care OR perinatal care OR postnatal care OR prenatal education OR maternal child AND Health Promotion OR Program OR Health Education OR Primary Prevention OR Immunization) AND (community health services OR community health nursing OR home care services OR community).

#### Search sources

The literature search was completed between May 2019 and July 2019. Table 1 illustrates the search sources for the peer-reviewed and grey literature for the review. Grey literature consisted of materials related to the review aims, such as reports and websites, not published from commercial organizations that typically produce peer-reviewed literature [41]. Based on the librarian’s advice, we completed a focused grey literature search of targeted sources [41]. Grey literature sources were determined through consultation with the librarian and reference lists from included articles, as suggested in other literature reviews [4, 42]. See Additional file 1 for a search example.

**Table 1:** Literature search sources

Scholarly databases	Grey literature sources	Other
Ovid MEDLINE EBSCO CINAHL Embase Scopus	Public Health Agency of Canada Health Canada Public Health Agency of Canada Best Practices Portal Aboriginal Ways Tried and True National Aboriginal Health Organization National Collaborating Centre for Aboriginal Health Indigenous Services Canada – Indigenous Health iPortal Indigenous Studies Portal Research Tool	Reference lists of included articles

#### Stage 3: study selection

##### Inclusion and exclusion criteria

Following the process in Arksey and O’Malley [21], the review team established the inclusion and exclusion criteria to be applied to all citations identified in Stage 2. Inclusion criteria consisted of articles specific to Indigenous peoples; maternal-child health programs; children aged 0–6 years of age; prenatal mothers; postnatal mothers; primary prevention; located within the community; all types of studies and methods; English language; timeframe 1990-April 2019; programs implemented in the countries, Australia, Canada, New Zealand, and the United States. Exclusion criteria consisted of articles, not Indigenous-specific; children > 6 years of age; acute care-based (i.e., hospital); outside designated timeframe; focused on a specific program element (i.e., the development of a survey for the evaluation of a health program); epidemiological focused (i.e., incidence, prevalence); disease-based.

##### Screening process

**Fig. 1:**
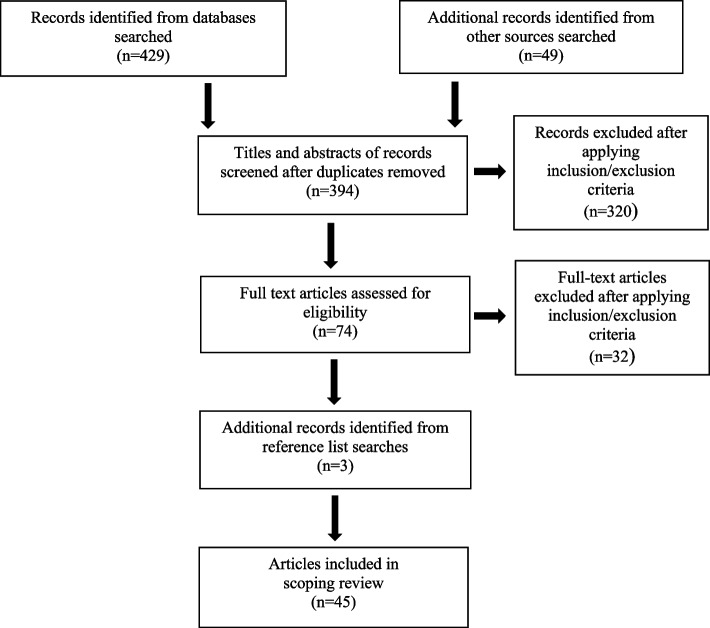
Screening process of selected articles

In August 2019, two of the three (CT, TM, and DT) reviewers independently screened each Title and abstract against the inclusion and exclusion criteria using the Rayyan software developed by Ouzzani et al. [43] Once title and abstract screening were complete, the full text articles were screened against the inclusion and exclusion criteria. As suggested by Levac et al. [20], a fourth reviewer (MS) was consulted to settle any disagreements between reviewers surrounding potential inclusions. Once the screening process was complete, 45 articles remained for inclusion in the scoping review including 36 peer-reviewed articles and 9 documents from grey literature. The screening process of selected articles can be found in Fig. 1.

#### Stage 4: charting the data

##### Data extraction from included literature

Following the Levac et al. [20] process, information was extracted from the 45 articles and summarized in a table. The data extraction table included: author, year, and title; Indigenous first author; country and population; methodology; aims/purpose; program description; reported factors of program success; reported program barriers; frontline worker role in health program process; and program outcomes. From August 2019 to October 2019, two reviewers (CT, TM, or DT) independently extracted data from each article and recorded it in a data extraction table. As suggested in Levac et al. [20], results were compared to determine consistency between reviewers. The data extraction tables can be found in Additional file 2.

#### Stage 5: collating, summarizing, and reporting the results

##### Data analysis methods

Analysis of the extracted data included descriptive statistics, a thematic analysis, and a Principal Component Analysis [PCA]. Descriptive statistics were compiled using the Social Sciences Statistical Package [SPSS] 25.0 from IBM Corp [44] to describe study characteristics and the role of frontline workers in the health program process.

##### Thematic analysis

For the thematic analysis portion of the scoping review, Levac et al. [20] recommend using a qualitative analysis technique. The review team chose the Braun and Clarke [45] thematic analysis framework to guide our analysis as this framework has been used extensively in the health field, including previous scoping reviews on health topics affecting Indigenous peoples [42, 46].

From November 2019 to January 2020, the Braun and Clarke [45] thematic analysis framework was applied by two authors (CT and TM) to the extracted data to generate codes and develop themes. The themes and corresponding codes and definitions supporting the themes were then distributed to all scoping review team members for review and refinement. No changes to the themes were requested by the team.

##### Principal component analysis

In an effort to address potential reviewer bias in identifying the themes in the extracted data (i.e., codes), a PCA using SPSS 25.0 [44] was completed by an independent reviewer (MS) to identify the themes (factors) underlying program success and barriers. The PCA was conducted with Promax oblique rotations applied to the codes from the thematic analysis. To identify the number of factors related to program success and barriers, a Parallel Analysis was performed (with 1000 Monte Carlo simulation repetitions). Codes that were moderately to strongly correlated to a factor (i.e., loadings –1.0 to –0.4 and 0.4 to 1.0) were used to name the themes.

## Results

### Characteristics of included literature

Reviewers (CT, TM, and DT) searched publicly available online biographies in an attempt to determine the self-situation or positionality [47] discussed by Kovach [48] of first authors of Indigenous related literature. For most articles the reviewers were unable to determine whether or not the first author self-identified as Indigenous. (*n* = 28). A self-identified Indigenous first author was determined for a small portion of articles only (*n* = 4). The majority of study designs (*n* = 16) within the literature were qualitative; followed by mixed methods (*n* = 12); quantitative (*n* = 8); program descriptions (*n* = 5); and literature reviews (*n* = 4). The majority of articles originated in Canada (*n* = 18) and Australia (*n* = 17); with the United States (*n* = 8); New Zealand (*n* = 1); and a combination of these countries (*n* = 1) rounding out the remaining articles.

### Factors of program success

From the thematic analysis using the Braun and Clarke [45] framework, seven themes were identified as contributing to program success for Indigenous maternal-child community health programs: relationship building; cultural; knowledge transmission styles; community collaboration; program approaches; staff; and operational considerations. Table 2 summarizes descriptions of the identified themes.

**Table 2:** Themes contributing to program success

Relationship Building
• Three types of relationships identified—staff-to-client, staff-to-staff, and community networks (other agencies, programs, and services) [1, 3, 5, 8, 13, 49–53] • Respect and trust are essential in any type of relationship building [5, 49, 54, 55] • Institutional supports such as investing time and funding are necessary for the relationship-building process [22, 29, 49, 56, 57]
**Cultural**
• “Culturally appropriate” programs were responsive to their participants and based the program on cultural elements such as the Medicine Wheel and Indigenous protective factors [8, 50, 58–60] • “Culturally based” programs included structural cultural adaptations, traditional approaches, and traditional ways such as traditional parenting and lifestyle practices [3, 4, 15, 28, 52, 55, 61] • Programs identifying with “cultural competency” described program providers as grounded in cultural competency through training [28, 29, 31, 62, 63] • Programs described as providing “culturally safe care” included workers reflecting on their privilege and positioning, the training and recruitment of workers, and discussing sensitive issues [14, 53, 62] • Local culture encompassed the inclusion of local protocols, such as prayer and ceremony within the program, and local cultural elements, such as the Medicine Wheel and artwork [8, 15, 52, 58, 64, 65] • Elders were included in the health program process and, specific to maternal-child programs, the involvement of senior community women [3, 14, 49, 59, 66–68] • Including both oral and written communication in the community language was important in the programs [15, 53, 62, 69–71]
**Knowledge Transmission Styles**
• For program delivery, applying oral traditions such as story (i.e., yarning) [5, 15, 28, 50] • Delivering visual program information in the form of pictures, visual aids or videos [22, 30, 53, 72, 73] • Utilizing communication styles that were easy to understand with no jargon and written in the community language 5,8,33,53,70) • Program advertising that applied unconventional methods using word of mouth and social media streams such as Facebook to advertise programs [1, 51]
**Community Collaboration**
• A range of community inclusion within the program process was used from community support and involvement to community ownership and self-determination [4, 13, 22, 29, 32, 56, 57, 68, 74] • Programs that identified community leadership, governance and self-determination demonstrated communities that took leadership and ownership over the programs themselves [1, 4, 15, 50, 58, 59, 69, 70]
**Program Approaches**
• Programs that are voluntary, eligible to all community members, and flexible [1, 3, 31, 49, 73, 75] • Family-led, holistic programs that include extended family members [5, 14, 15, 51, 59, 67, 76] • Programs focused on strengths-based approaches and building on participants’ assets [1, 5, 28, 55, 75, 77, 78] • Program incentives such as resources or gift packs for participants [22, 54, 68, 76] • Home visits and leaving the clinic setting to bring the program to the client [5, 22, 50, 53, 77]
**Staff**
• Employing Indigenous staff [5, 15, 28, 49, 50, 60, 66, 67, 69] • Employing male staff [61, 72] • Staff that are long-term [1, 55, 60, 61, 71] • Staff-to-staff knowledge exchange with two-way sharing between cultures, i.e., Indigenous and non-Indigenous [5, 13, 52] • Clearly defined staff roles [29, 52]
**Operational Considerations**
• Resources such as adequate and long-term funding [3, 32, 63] • Welcoming physical space [1, 13, 29, 56, 57, 61, 66] • Provide transportation for participants [1, 8, 50, 67] • Organizational considerations within program operations such as support and training for staff [5, 13, 15, 49, 54, 61, 62, 69, 72] • Leadership and management that supports workers and collaborative approaches [8, 15, 22, 50, 52, 54, 72] • Policies at both the local level and beyond that support the work with families [14, 62] • Ongoing evaluation and improvement of programs that reflect the community and community priorities [55–57, 78]

### Program barriers

The thematic analysis using the Braun and Clarke [44, 45] framework identified six themes contributing to program barriers: impacts of colonization; power and governance; client and community barriers to accessing the program; physical and geographical challenges; staff; and operational deficits. Summary descriptions of the themes are found in Table 3.

**Table 3:** Themes contributing to program barriers

Impacts of Colonization
• Historical experiences such as forced removal of children and residential school experiences creating a lack of trust, fear, and trauma that impacts the ability to deliver programs in the present day [1, 49, 69, 73, 74] • Colonization impacts manifest in poverty, adverse living conditions such as gangs, violence, substance abuse, overcrowded housing, and low education resulting in low resources and poor health [1, 5, 15, 31, 60, 65, 68, 70, 77] • Infrastructure and policies do not support Indigenous family practices, creating a gap in care structure for Indigenous families [50, 53, 65]
**Power and Governance**
• Power imbalances were present in three domains client-to-staff, staff-to-staff, and program-to-community [52, 61, 64, 71] • Power imbalances are illustrated through mandated programs, cultural knowledge extraction, the exclusion of traditional knowledge from program decision-making, and struggling to balance worldviews [1, 4, 31, 52, 59, 69] • Formal oral and written information delivery systems are employed within the program [28, 31, 52] • The lack of community-specific research creates information gaps in program design [59, 63] • Jurisdictional issues between levels of government create funding inequities and impede program delivery [3, 63, 69, 71]
**Client and Community Barriers to Accessing the Program**
• From the client perspective, the program excludes family members; associated with stigma and high-risk participant criteria; provide inaccurate program information; offered in an unwelcoming physical space; experience with the child welfare system can create a lack of trust and deterrent from accessing available programs [1, 14, 15, 30, 61, 66, 68, 69, 77] • From the staff perspective, the beliefs and practices of a community are omitted in the program such as the norm of bottle-feeding versus breastfeeding and gender roles; clients suffering from mental health challenges such as depression; participants’ other obligations in the community such as demands of mothers; and client moving out of the program area [1, 22, 28, 30, 54, 71, 73, 75]
**Physical and Geographical Challenges**
• Remote and rural locations create access challenges, program availability, and delivery challenges within the community [5, 15, 32, 49, 54, 69, 70, 73] • Elements such as weather, transportation, and roads can inhibit staff trying to reach clients and clients attempting to reach the program [15, 49, 69]
**Staff**
• Staffing challenges that include understaffing, limited male staff, and very few Indigenous staff [15, 68, 77] • High staff workload and staff assuming multiple roles resulting in high staff burnout and turnover [32, 49, 53, 54, 64, 69, 71] • Staff safety concerns, such as domestic violence, crime, and dogs [3, 5, 31, 49] • Challenges between staff such as non-Indigenous staff racism, worker expertise not recognized, and the exclusion of team members [15, 31, 52, 61, 69] • Staff members may be resistant to a program [64, 65] • Staff lacks the necessary training resulting in the program not delivered as intended [31, 53, 68, 69, 71, 76] • Staff from the local community may face unique barriers not experienced by external staff, such as cultural and kinship barriers with close relationships to community members and role conflict between what the staff member must do to carry out the program (i.e., home visits) and the community norms [5, 31, 77] • Some program staff lacks cultural competence, which negatively impacts the client and the program delivery [29, 52, 61, 63]
**Operational Deficits**
• Lack of available space and technology (i.e., computers) to deliver the program or staff to complete their work [52, 64] • Time and resource constraints in the form of inadequate time and funding to build relationships, meet program demands, create program materials, and foster program sustainability [30, 33, 51, 52, 55, 69, 73] • Fractured service networks prevented some programs from coordinating with other agencies and services, which limited the program’s reach, i.e., not extending beyond program clients to the wider community [3, 32, 49, 60, 61, 76] • Policies and practices such as paperwork, disconnect of priorities between program and external organizations and departments and meeting the funding body's requirements [32, 49, 63, 68, 69, 76, 79] • Challenges to evaluating the program such as inadequate resources and the capacity to complete program evaluations with issues to available data, data management, and evaluation designs [3, 8, 15, 29, 55, 71, 75]

### Principal component analysis

The PCA yielded three key factors underlying program success: relationship; program implementation; and operational delivery. The PCA identified five overarching factors relating to program barriers: colonization and its impact; interpersonal staffing issues (issues amongst the staff); staff issues resulting from lack of cultural sensitivity and a lack of resources; challenges with how programs are being implemented; and access to programs. Although the thematic analysis and PCA were completed independently, the results of the PCA illustrate themes similar to those identified through the thematic analysis.

### Role of frontline workers

A large portion of the reviewed literature (*n* = 29) did not explicitly state the role of frontline workers outside of program delivery. Program descriptions provided an alternative means for the reviewers to possibly identify the role of frontline workers. Within the reviewed articles (*n* = 45), frontline workers all had a role in program delivery. For a majority of the articles (*n* = 25), we could not determine if frontline workers had been involved in program development; less than half of frontline workers (*n* = 19) had a role in program development; and one article (*n* = 1) stated no involvement of frontline workers in program development. For the largest portion of the articles (*n* = 38), we could not determine if frontline workers were involved in developing the program evaluations, i.e., determining the evaluation design, methods, and measures of success; very few frontline workers (*n* = 6) had a role in developing program evaluations; and one article (*n* = 1) reported no involvement of frontline workers in developing the program evaluation. In most of the literature (*n* = 20), we could not determine if frontline workers participated in the program evaluations; in approximately half of the reviewed literature (*n* = 22), frontline workers participated in program evaluation; and a small portion of articles (*n* = 3) reported no frontline workers participating in the evaluations.

## Discussion

### The importance of authorship

Self-situation or positionality conveys who the author is and how the author's perspective shapes the research [17, 47, 48]. The lack of positionality of authors, makes it challenging to identify the voices that are communicating research in Indigenous maternal-child health. The voices sharing the findings of Indigenous maternal-child health program research are important to research consumers because they can influence how data is analyzed, interpreted, and communicated to inform practice [17, 47, 48, 80].

In a large portion of articles (*n* = 28) included in this scoping review, the reviewers were unable to determine the self-situation or positionality [47, 48] of first authors. Currently, publication guidelines and length limitations may not permit researchers to describe their background and motivation for the project [35, 48, 81]. One way to strengthen the literature and research for consumers is to include authors’ positionality in the literature when communicating Indigenous health research [48]. Including positionality could help decolonize the peer-reviewed literature by creating space for an Indigenous perspective and potentially influencing what research is translated into practice [48, 82]. Changing the peer-reviewed literature may help discontinue the cycle of knowledge used in decision-making that perpetuates colonial health policy and practices that have done little to reduce Indigenous health inequity [79, 82, 83].

### Factors of program success and barriers

#### Connection between the factors of success and program barriers

There appears to be a connection between the themes or those factors important for program success and ones acting as barriers; efforts to include elements for success may also help to address a program barrier. The connectivity between themes could be used as levers to strengthen programs. For example, the inclusion of extended families within the program approach has been identified as contributing to program success [1, 4, 15, 49, 77]. Incorporating extended families into a maternal-child health program may help address an identified barrier to accessing the program, such as the exclusion of fathers [5, 8, 66, 77]. The thematic results reveal linkages between factors of program success and barriers that provide insight into areas and strategies that could be used to improve the health program process, i.e., program development and evaluation, and quality improvement. Application of these findings may positively impact Indigenous maternal-child health programs to increase program success.

#### Culture and maternal-child health programs

Our scoping review results highlight that culture does not stand alone as an identified factor of maternal-child health program success, but is interwoven throughout the themes, from the inclusion of local culture to knowledge transmission styles [14, 50, 52, 53, 65, 73]. Although woven through the themes, in the reviewed literature there is a lack of acknowledgement or discussion of culture’s importance as an intervention that is emphasized by Sasakamoose et al. [84]. The discourse surrounding culture in the literature focuses on the inclusion of culture within programs, such as Indigenous artwork, story, kinship systems, or cultural terms to describe the program, such as culturally-based, culturally-appropriate, and culturally-safe care [3, 5, 15, 28, 53, 68]. Cultural inclusion within the programs is intended to create an acceptable program for participants and a good fit for the community [15, 55, 65].

The lack of discussion surrounding Indigenous culture as an intervention may suggest that the full benefit and impact of culture as a tool for wellness [84] are not being realized within Indigenous maternal-child health programs. A knowledge gap within health program literature exists; it is not well understood that Indigenous culture brings strengths and protective health benefits to the program itself to foster positive health outcomes and reduce health inequity [4, 84]. Omitting the acknowledgement and discussion of culture as an intervention creates a missed opportunity to engage in reconciliation and decolonization of programs [84]. 

### Essential role of program staff in Indigenous maternal-child health programs

The literature included in this review identified the impact of staff on the program as either positive or negative [1, 5, 52, 53, 61, 62, 72], demonstrating a large part of program success and challenges are dependent on the people within the program. For example, successful relationships with program participants can contribute to program success and are primarily dependent on individual staff [5, 8, 52]. The Indigenous program staff was identified in half of the reviewed literature (*n* = 23) as essential to providing culturally competent and culturally safe care for program participants and contributing to program success [15, 52, 62, 67]. The literature discusses staff characteristics, such as valuing relationships, displaying genuine empathy, and being respectful, as important for creating successful Indigenous maternal health programs [8, 29, 77]. Conversely, staff who lack cultural competence and are resistant to programs can create significant program challenges and may result in clients not accessing a program or not receiving the full benefit of the program [29, 61, 64, 65].

The literature identifies that staff have an important role in Indigenous maternal-child health programs [5, 52, 60]. Hence staffing and hiring practices are areas that may impact program success and failures. Union-based work environments can create challenges to hiring practices that support the employment of Indigenous staff and individuals who possess characteristics that make them a good fit for maternal-child health programs [25, 77, 85]. For example, hiring may only be available to existing staff through internal union opportunities where seniority is a primary factor in awarding positions and determining successful candidates [86, 87]. These limitations on hiring processes can create significant challenges to implementing best practices in recruiting providers for Indigenous maternal-child health programs. To bring best practice recommendations surrounding staffing forward requires employers and unions to work together and further develop hiring practices [88] that facilitate program success.

### Frontline worker role in Indigenous maternal-child health programs

With the essential role of staff in maternal-child health programs [4, 5, 15], there is great potential for frontline workers to take an extended role in the health program process, beyond their primary role in program delivery. Although almost half of the reviewed articles reported frontline workers participating in program development, frontline workers can offer more to health program development than the current practice found in our review that frames their contributions exclusively on providing input on methods of program delivery and adapting program resources [5, 15, 67].

Frontline workers have local community knowledge and relationships within the community [4, 8, 77]. They provide an exclusive perspective that can be used to identify community-specific needs, set health program priorities, and assume a role in program evaluation [4, 5, 8, 77]. Frontline workers are part of the community in which programs are delivered and can provide valuable contributions to all aspects of the health program process, i.e., development, planning, delivery, and evaluation [4, 12, 15]. Providing frontline workers with the opportunity of inclusion to share their knowledge and skills can: increase the likelihood of programs that align with local community values and practices; increase program relevance to the community; and, potentially, contribute to program success and create a positive impact on health outcomes [4, 8, 52].

#### Stage 6: consultation

Consultation with stakeholders is part of the scoping review process as discussed by Arksey and O’Malley [21] and Levac et al. [20]. The inclusion of stakeholders and their input may help to inform the review, strengthen the review’s findings, and provide direction for future research [20, 21]. In addition, the inclusion of stakeholders is an essential and ethical responsibility in research that may impact practice in Indigenous health [20, 89]. Central to a completed community-based participatory research project was accessing the perspective of our community partner to explore the scoping review research questions from the perspectives of stakeholders such as maternal-child health program families, frontline workers, and administrators. The findings from the scoping literature review were applied to inform the methodology and discussion of the community-based research project and identify convergences or divergences between the stakeholder perspective and reviewed literature.

## Limitations

Some articles and programs may have been missed due to the search strategy employed. The data extraction relied on program descriptions from the included articles and there is potential that relevant data may have been missed as it was not included in the article [4]. Determining the self-situation of first authors depended on the retrieved literature and seeking out the authors’ publicly available biographies. These methods may not have been adequate to determine the published first author’s self-situation resulting in some authors being misidentified or missed.

Although the authors attempted to reduce bias by including (1) two reviewers in the study selection and data extraction and (2) a PCA in addition to the Braun and Clarke [45] thematic analysis, the potential for bias within the review remains. The review was limited to four countries: Australia, Canada, New Zealand, and the United States, based on similar histories of colonization and large Indigenous populations [34, 35] therefore, the applicability of the results outside of these countries is unknown. Even within the countries of inclusion, Indigenous peoples, communities, and cultures are all distinct with local values, practices, and protocols [7]. The scoping review results may not apply to all Indigenous maternal-child health programs within the included countries.

## Conclusion

This scoping review provided an overview of the literature about factors of Indigenous maternal-child health program success, barriers, and the role of frontline workers. Although a better understanding of the frontline worker role in maternal-child health programs was obtained from the review, there was a large proportion of literature where the authors could not determine if the role went beyond program delivery. In addition, no direct input from frontline workers and their perspectives on program success or barriers were identified, suggesting areas to explore in future research. Although the researchers hypothesized the strong connection between frontline workers and maternal-child health programs, one unanticipated finding from the review was the “loud” nature of the literature supporting the importance of staff in health programs. The findings from this scoping review have informed the methodology and analysis of a community-based participatory research project. Outside of the study, the review’s findings may help improve the development, delivery, and evaluation of Indigenous maternal-child health programs.

### Supplementary Information

**Additional file 1. **Example of online database search – OVID Medline.

**Additional file 2.**

## Data Availability

The datasets supporting the conclusions of this article are included within the article and in Additional file 1.
