# Supplementary material for: Factors of success, barriers, and the role of frontline workers in Indigenous maternal-child health programs: a scoping review
Source: Int J Equity Health. 2024 Feb 13;23:28. doi: 10.1186/s12939-024-02118-2 (PMC10863162; doi:10.1186/s12939-024-02118-2)
Supplement: Supplementary file 1 — Additional file 1. Example of online database search – OVID Medline. [file 12939_2024_2118_MOESM1_ESM.docx]

Additional File 1. Example of online database search – OVID Medline

|  |  |
| --- | --- |

# OVID: Search Form

Top of Form

## Search History

(20searches found)

| |  | **[# ▼](http://ovidsp.dc2.ovid.com.cyber.usask.ca/sp-3.33.0b/ovidweb.cgi?&S=KBLNFPFCGEEBJALKJPDKFGBHJCCIAA00&Sort+Sets=ascending)** | **Searches** | **Results** | **Type** | | --- | --- | --- | --- | --- | |  | 20 | 18 and 19 | 540 | Advanced | |  | 19 | 1 or 2 or 3 or 4 or 5 | 50860 | Advanced | |  | 18 | 16 and 17 | 31297 | Advanced | |  | 17 | 10 or 11 or 12 or 13 or 14 or 15 | 3043550 | Advanced | |  | 16 | 6 or 7 or 8 or 9 | 130345 | Advanced | |  | 15 | maternity.mp. | 18999 | Advanced | |  | 14 | Prenatal Care/ or prenatal.mp. or Prenatal Education/ | 155116 | Advanced | |  | 13 | maternal health program.mp. | 20 | Advanced | |  | 12 | Infant/ or Infant, Newborn/ | 1095002 | Advanced | |  | 11 | Maternal Health/ or Pregnancy/ | 844721 | Advanced | |  | 10 | Maternal-Child Health Services/ or Child/ or Child Health/ or Child, Preschool/ or Child Health Services/ | 1833789 | Advanced | |  | 9 | prevention program.mp. | 6121 | Advanced | |  | 8 | Health Education/ | 58888 | Advanced | |  | 7 | health program.mp. | 4654 | Advanced | |  | 6 | Health Promotion/ | 69524 | Advanced | |  | 5 | native american.mp. | 3071 | Advanced | |  | 4 | aboriginal.mp. | 7173 | Advanced | |  | 3 | american indian.mp. or Indians, North American/ | 14921 | Advanced | |  | 2 | Oceanic Ancestry Group/ or indigenous.mp. | 32246 | Advanced | |  | 1 | (exp Indians, North American/ or exp Inuits/ or exp Health Services, Indigenous/ or exp Ethnopharmacology/ or Athapaskan.mp. or Saulteaux.mp. or Wakashan.mp. or Cree.mp. or Dene.mp. or Inuit.mp. or Inuk.mp. or Inuvialuit*.mp. or Haida.mp. or Ktunaxa.mp. or Tsimshian.mp. or Gitsxan.mp. or Nisga'a.mp. or Haisla.mp. or Heiltsuk.mp. or Oweenkeno.mp. or Kwakwaka'wakw.mp. or Nuu chah nulth.mp. or Tsilhqot'in.mp. or Dakelh.mp. or Wet'suwet'en.mp. or Sekani.mp. or Dunne-za.mp. or Dene.mp. or Tahltan.mp. or Kaska.mp. or Tagish.mp. or Tutchone.mp. or Nuxalk.mp. or Salish.mp. or Stl'atlimc.mp. or Nlaka'pamux.mp. or Okanagan.mp. or Sec wepmc.mp. or Tlingit.mp. or Anishinaabe.mp. or Blackfoot.mp. or Nakoda.mp. or Tasttine.mp. or Tsuu T'inia.mp. or Gwich'in.mp. or Han.mp. or Tagish.mp. or Tutchone.mp. or Algonquin.mp. or Nipissing.mp. or Ojibwa.mp. or Potawatomi.mp. or Innu.mp. or Maliseet.mp. or Mi'kmaq.mp. or Micmac.mp. or Passamaquoddy.mp. or Haudenosaunee.mp. or Cayuga.mp. or Mohawk.mp. or Oneida.mp. or Onodaga.mp. or Seneca.mp. or Tuscarora.mp. or Wyandot.mp. or Aboriginal*.mp. or Indigenous*.mp. or Metis.mp. or red road.mp. or "on reserve".mp. or off-reserve.mp. or First Nation.mp. or First Nations.mp. or Amerindian.mp. or (urban adj3 (Indian* or Native* or Aboriginal*)).mp. or ethnomedicine.mp. or country food*.mp. or residential school*.mp. or ((exp Medicine, Traditional/ or traditional medicine*.mp.) not Chinese.mp.) or exp Shamanism/ or shaman*.mp. or traditional heal*.mp. or traditional food*.mp. or medicine man.mp. or medicine woman.mp. or autochtone*.mp. or (Native* adj1 (man or men or women or woman or boy* or girl* or adolescent* or youth or youths or person* or adult or people* or Indian* or Nation or tribe* or tribal or band or bands)).mp.) and (exp Canada/ or (Canad* or British Columbia or Columbie Britannique or Alberta or Saskatchewan or Manitoba or Ontario or Quebec or Nova Scotia or New Brunswick or Newfoundland or Labrador or Prince Edward Island or Yukon Territory or NWT or Northwest Territories or Nunavut or Nunavik or Nunatsiavut or NunatuKavut)).mp. | 6147 | Advanced | | Combine with: | | |  | | [Contract](http://ovidsp.dc2.ovid.com.cyber.usask.ca/sp-3.33.0b/ovidweb.cgi?&S=KBLNFPFCGEEBJALKJPDKFGBHJCCIAA00&SELECT=S.sh%7c&Contract=1&Main+Search+Page=Main+Search+Page) |
| --- | --- | --- | --- | --- | --- | --- | --- | --- | --- | --- | --- | --- | --- | --- | --- | --- | --- | --- | --- | --- | --- | --- | --- | --- | --- | --- | --- | --- | --- | --- | --- | --- | --- | --- | --- | --- | --- | --- | --- | --- | --- | --- | --- | --- | --- | --- | --- | --- | --- | --- | --- | --- | --- | --- | --- | --- | --- | --- | --- | --- | --- | --- | --- | --- | --- | --- | --- | --- | --- | --- | --- | --- | --- | --- | --- | --- | --- | --- | --- | --- | --- | --- | --- | --- | --- | --- | --- | --- | --- | --- | --- | --- | --- | --- | --- | --- | --- | --- | --- | --- | --- | --- | --- | --- | --- | --- | --- | --- | --- | --- |

|  |
| --- |
| Create RSS    Name  Comment    Comment |

Bottom of Form

Top of Form

- [Basic Search](http://ovidsp.dc2.ovid.com.cyber.usask.ca/sp-3.33.0b/ovidweb.cgi?S=KBLNFPFCGEEBJALKJPDKFGBHJCCIAA00&Display+Mode=easy)
- [Find Citation](http://ovidsp.dc2.ovid.com.cyber.usask.ca/sp-3.33.0b/ovidweb.cgi?S=KBLNFPFCGEEBJALKJPDKFGBHJCCIAA00&Display+Mode=findcite)
- [Search Tools](http://ovidsp.dc2.ovid.com.cyber.usask.ca/sp-3.33.0b/ovidweb.cgi?S=KBLNFPFCGEEBJALKJPDKFGBHJCCIAA00&Display+Mode=subjsrch)
- [Search Fields](http://ovidsp.dc2.ovid.com.cyber.usask.ca/sp-3.33.0b/ovidweb.cgi?S=KBLNFPFCGEEBJALKJPDKFGBHJCCIAA00&Display+Mode=fields)
- Advanced Search
- [Multi-Field Search](http://ovidsp.dc2.ovid.com.cyber.usask.ca/sp-3.33.0b/ovidweb.cgi?S=KBLNFPFCGEEBJALKJPDKFGBHJCCIAA00&Display+Mode=multifield)

1 Resource selected | [Hide](http://ovidsp.dc2.ovid.com.cyber.usask.ca/sp-3.33.0b/ovidweb.cgi?&S=KBLNFPFCGEEBJALKJPDKFGBHJCCIAA00&Display+Mode=ovidclassic&Get+Resources+Widget=0) | [Change](http://ovidsp.dc2.ovid.com.cyber.usask.ca/sp-3.33.0b/ovidweb.cgi?&S=KBLNFPFCGEEBJALKJPDKFGBHJCCIAA00&Change+Database=1)

| [Database Field Guide](http://ovidsp.dc2.ovid.com.cyber.usask.ca/sp-3.33.0b/ovidweb.cgi?&S=KBLNFPFCGEEBJALKJPDKFGBHJCCIAA00&Database+Field+Guide=9) Ovid MEDLINE(R) 1946 to May Week 4 2019 |
| --- |

Search by Keyword Author Title Journal

Enter keyword or phrase (* or $ for truncation)

Enter Author's last name, a space, and first initial if known

Enter title word or phrase to search.

Enter the first few letters of a full journal name; no abbreviations

Include Multimedia Map Term to Subject Heading

Limits  (expand) (close)

| Abstracts | Structured Abstracts | English Language |
| --- | --- | --- |
| Ovid Full Text Available | Review Articles | Humans |
| Core Clinical Journals (AIM) | Latest Update | Pharmacologic Actions |

| Publication Year Publication Year- |  |
| --- | --- |

Status

Bottom of Form

To search Open Access content on Ovid, go to [Basic Search.](http://ovidsp.dc2.ovid.com.cyber.usask.ca/sp-3.33.0b/ovidweb.cgi?&S=KBLNFPFCGEEBJALKJPDKFGBHJCCIAA00&Display+Mode=easy)

Top of Form

## Results Tools

[Optionsopens a popup window](http://ovidsp.dc2.ovid.com.cyber.usask.ca/sp-3.33.0b/ovidweb.cgi?&S=KBLNFPFCGEEBJALKJPDKFGBHJCCIAA00&RT+Widget+Options=RT)

[search_left_heading] text for en not available

## Search Information

You searched:

18 and 19

- + Search terms used:

- aboriginal
- aboriginal*
- adolescent*
- adult
- alberta
- algonquin
- american
- indian
- amerindian
- anishinaabe
- athapaskan
- autochtone*
- band
- bands
- blackfoot
- boy*
- british
- columbia
- canad*
- canada
- cayuga
- child
- health
- services
- child,
- preschool
- columbie
- britannique
- country
- food*
- cree
- dakelh
- dene
- dunne-za
- ethnomedicine
- ethnopharmacology
- first
- nation
- nations
- girl*
- gitsxan
- gwich'in
- haida
- haisla
- han
- haudenosaunee
- education
- program
- promotion
- services,
- indigenous
- heiltsuk
- indian*
- indians,
- north
- indigenous*
- infant
- infant,
- newborn
- innu
- inuit
- inuits
- inuk
- inuvialuit*
- kaska
- ktunaxa
- kwakwaka'wakw
- labrador
- maliseet
- man
- manitoba
- maternal
- maternal-child
- maternity
- medicine
- woman
- medicine,
- traditional
- men
- metis
- mi'kmaq
- micmac
- mohawk
- nakoda
- native
- native*
- new
- brunswick
- newfoundland
- nipissing
- nisga'a
- nlaka'pamux
- northwest
- territories
- nova
- scotia
- nunatsiavut
- nunatukavut
- nunavik
- nunavut
- nuu
- chah
- nulth
- nuxalk
- nwt
- oceanic
- ancestry
- group
- off-reserve
- ojibwa
- okanagan
- on
- reserve
- oneida
- onodaga
- ontario
- oweenkeno
- passamaquoddy
- people*
- person*
- potawatomi
- pregnancy
- prenatal
- care
- prevention
- prince
- edward
- island
- quebec
- red
- road
- residential
- school*
- salish
- saskatchewan
- saulteaux
- sec
- wepmc
- sekani
- seneca
- shaman*
- shamanism
- stl'atlimc
- tagish
- tahltan
- tasttine
- tlingit
- heal*
- medicine*
- tribal
- tribe*
- tsilhqot'in
- tsimshian
- tsuu
- t'inia
- tuscarora
- tutchone
- urban
- wakashan
- wet'suwet'en
- women
- wyandot
- youth
- youths
- yukon
- territory

**Search Returned:**
540 text results

**Sort By:**

[Customize Display opens a popup window](http://ovidsp.dc2.ovid.com.cyber.usask.ca/sp-3.33.0b/ovidweb.cgi?&S=KBLNFPFCGEEBJALKJPDKFGBHJCCIAA00&Customize+Display=S.sh.79)

## Filter By

[Add to Search History](http://ovidsp.dc2.ovid.com.cyber.usask.ca/sp-3.33.0b/ovidweb.cgi?&S=KBLNFPFCGEEBJALKJPDKFGBHJCCIAA00&Add+Search+Strategy=S.sh.79%7cS.sh.79.80)

 ( 0 )

- Years
  All Years
  - [Current year](http://ovidsp.dc2.ovid.com.cyber.usask.ca/sp-3.33.0b/ovidweb.cgi?&S=KBLNFPFCGEEBJALKJPDKFGBHJCCIAA00&Process+Filter=S.sh.79%7cS.sh.79.80%7c0&filter_id=780&filterset=Date)
  - [Past 3 years](http://ovidsp.dc2.ovid.com.cyber.usask.ca/sp-3.33.0b/ovidweb.cgi?&S=KBLNFPFCGEEBJALKJPDKFGBHJCCIAA00&Process+Filter=S.sh.79%7cS.sh.79.80%7c0&filter_id=781&filterset=Date)
  - [Past 5 years](http://ovidsp.dc2.ovid.com.cyber.usask.ca/sp-3.33.0b/ovidweb.cgi?&S=KBLNFPFCGEEBJALKJPDKFGBHJCCIAA00&Process+Filter=S.sh.79%7cS.sh.79.80%7c0&filter_id=782&filterset=Date)
  - Specific Year Range
- Subject
- Author
- Journal
- Publication Type

## My Projects

[+ New Project](http://ovidsp.dc2.ovid.com.cyber.usask.ca/sp-3.33.0b/ovidweb.cgi?&S=KBLNFPFCGEEBJALKJPDKFGBHJCCIAA00&FormProcessor=project__add&InputArgs=form_name%3aproject__add%7ckeep_selection%3aY%7cdisplay_type%3aTitles+Display%7cdlname%3aS.sh.79%7cstart%3aC%7ccount%3a%7cgsrd_num%3a)

No projects available.

# Multimedia

- Print
- Email
- Export
- Add to My Projects
- [Keep Selected](http://ovidsp.dc2.ovid.com.cyber.usask.ca/sp-3.33.0b/ovidweb.cgi?&S=KBLNFPFCGEEBJALKJPDKFGBHJCCIAA00&View+Selected+Results=1)

All


**The number of results you have selected is exceeding your institution limits.**

|  | 1. | [A Pragmatic Review to Assist Planning and Practice in Delivering Nutrition Education to Indigenous Youth. [Review]](http://ovidsp.dc2.ovid.com.cyber.usask.ca/sp-3.33.0b/ovidweb.cgi?&S=KBLNFPFCGEEBJALKJPDKFGBHJCCIAA00&Complete+Reference=S.sh.79%7c1%7c1&Counter5=SS_view_found_complete%7c30818853%7cmesz%7cmedline%7cmedl&Counter5Data=30818853%7cmesz%7cmedline%7cmedl)  Kagie R; Lin SN; Hussain MA; Thompson SC.  Nutrients. 11(3), 2019 Feb 27.  [Journal Article. Review]  UI: 30818853  Authors Full Name Kagie, Robin; Lin, Szu-Yu Nancy; Hussain, Mohammad Akhtar; Thompson, Sandra C.    [My Projects](http://ovidsp.dc2.ovid.com.cyber.usask.ca/sp-3.33.0b/ovidweb.cgi?&S=KBLNFPFCGEEBJALKJPDKFGBHJCCIAA00&FormProcessor=item__add&InputArgs=form_name%3aitem__add%7cinterface_origin%3aoi%7ckeep_selection%3aY%7cdisplay_type%3aTitles%7cdlname%3aS.sh.79%7cstart%3a1%7ccount%3a10%7cindex%3a1%7con_mm_page%3a%7c&Counter5=SS_add_to_my_projects%7c30818853%7cmesz%7cmedline%7cmedl) [Annotate](http://ovidsp.dc2.ovid.com.cyber.usask.ca/sp-3.33.0b/ovidweb.cgi?&S=KBLNFPFCGEEBJALKJPDKFGBHJCCIAA00&Titles=S.sh.79%7c1%7c10&Annotate=S.sh.79%7c1%7c0&IECachePoison=27621834.1101721&Counter5=SS_annotate%7c30818853%7cmesz%7cmedline%7cmedl&Counter5Sessionless=1) |
| --- | --- | --- |

|  | 2. | [Aboriginal youth's perceptions of traditional and commercial tobacco in Canada.](http://ovidsp.dc2.ovid.com.cyber.usask.ca/sp-3.33.0b/ovidweb.cgi?&S=KBLNFPFCGEEBJALKJPDKFGBHJCCIAA00&Complete+Reference=S.sh.79%7c2%7c1&Counter5=SS_view_found_complete%7c28973397%7cmesz%7cmedline%7cmedl&Counter5Data=28973397%7cmesz%7cmedline%7cmedl)  Gendron F.  Health Promotion International. 33(6):1033-1041, 2018 Dec 01.  [Comparative Study. Journal Article]  UI: 28973397  Authors Full Name Gendron, Fidji.    [My Projects](http://ovidsp.dc2.ovid.com.cyber.usask.ca/sp-3.33.0b/ovidweb.cgi?&S=KBLNFPFCGEEBJALKJPDKFGBHJCCIAA00&FormProcessor=item__add&InputArgs=form_name%3aitem__add%7cinterface_origin%3aoi%7ckeep_selection%3aY%7cdisplay_type%3aTitles%7cdlname%3aS.sh.79%7cstart%3a1%7ccount%3a10%7cindex%3a2%7con_mm_page%3a%7c&Counter5=SS_add_to_my_projects%7c28973397%7cmesz%7cmedline%7cmedl) [Annotate](http://ovidsp.dc2.ovid.com.cyber.usask.ca/sp-3.33.0b/ovidweb.cgi?&S=KBLNFPFCGEEBJALKJPDKFGBHJCCIAA00&Titles=S.sh.79%7c1%7c10&Annotate=S.sh.79%7c2%7c0&IECachePoison=99303110.8560068&Counter5=SS_annotate%7c28973397%7cmesz%7cmedline%7cmedl&Counter5Sessionless=1) |  |
| --- | --- | --- | --- |

|  | 3. | [Fidelity of motivational interviewing in an American Indian oral health intervention.](http://ovidsp.dc2.ovid.com.cyber.usask.ca/sp-3.33.0b/ovidweb.cgi?&S=KBLNFPFCGEEBJALKJPDKFGBHJCCIAA00&Complete+Reference=S.sh.79%7c3%7c1&Counter5=SS_view_found_complete%7c29461622%7cmesz%7cmedline%7cmedl&Counter5Data=29461622%7cmesz%7cmedline%7cmedl)  Wilson AR; Fehringer KA; Henderson WG; Venner K; Thomas J; Harper MM; Batliner TS; Albino J.  Community Dentistry & Oral Epidemiology. 46(3):310-316, 2018 06.  [Journal Article. Randomized Controlled Trial. Research Support, N.I.H., Extramural]  UI: 29461622  Authors Full Name Wilson, Anne R; Fehringer, Karen A; Henderson, William G; Venner, Kamilla; Thomas, Jacob; Harper, Maya M; Batliner, Terrence S; Albino, Judith.    [My Projects](http://ovidsp.dc2.ovid.com.cyber.usask.ca/sp-3.33.0b/ovidweb.cgi?&S=KBLNFPFCGEEBJALKJPDKFGBHJCCIAA00&FormProcessor=item__add&InputArgs=form_name%3aitem__add%7cinterface_origin%3aoi%7ckeep_selection%3aY%7cdisplay_type%3aTitles%7cdlname%3aS.sh.79%7cstart%3a1%7ccount%3a10%7cindex%3a3%7con_mm_page%3a%7c&Counter5=SS_add_to_my_projects%7c29461622%7cmesz%7cmedline%7cmedl) [Annotate](http://ovidsp.dc2.ovid.com.cyber.usask.ca/sp-3.33.0b/ovidweb.cgi?&S=KBLNFPFCGEEBJALKJPDKFGBHJCCIAA00&Titles=S.sh.79%7c1%7c10&Annotate=S.sh.79%7c3%7c0&IECachePoison=93410633.317254&Counter5=SS_annotate%7c29461622%7cmesz%7cmedline%7cmedl&Counter5Sessionless=1) |  |
| --- | --- | --- | --- |

|  | 4. | [Is culturally based prevention effective? Results from a 3-year tribal substance use prevention program.](http://ovidsp.dc2.ovid.com.cyber.usask.ca/sp-3.33.0b/ovidweb.cgi?&S=KBLNFPFCGEEBJALKJPDKFGBHJCCIAA00&Complete+Reference=S.sh.79%7c4%7c1&Counter5=SS_view_found_complete%7c30092491%7cmesz%7cmedline%7cmedl&Counter5Data=30092491%7cmesz%7cmedline%7cmedl)  Kelley A; Fatupaito B; Witzel M.  Evaluation & Program Planning. 71:28-35, 2018 12.  [Journal Article. Multicenter Study. Research Support, U.S. Gov't, P.H.S.]  UI: 30092491  Authors Full Name Kelley, Allyson; Fatupaito, Bethany; Witzel, Morgan.    [My Projects](http://ovidsp.dc2.ovid.com.cyber.usask.ca/sp-3.33.0b/ovidweb.cgi?&S=KBLNFPFCGEEBJALKJPDKFGBHJCCIAA00&FormProcessor=item__add&InputArgs=form_name%3aitem__add%7cinterface_origin%3aoi%7ckeep_selection%3aY%7cdisplay_type%3aTitles%7cdlname%3aS.sh.79%7cstart%3a1%7ccount%3a10%7cindex%3a4%7con_mm_page%3a%7c&Counter5=SS_add_to_my_projects%7c30092491%7cmesz%7cmedline%7cmedl) [Annotate](http://ovidsp.dc2.ovid.com.cyber.usask.ca/sp-3.33.0b/ovidweb.cgi?&S=KBLNFPFCGEEBJALKJPDKFGBHJCCIAA00&Titles=S.sh.79%7c1%7c10&Annotate=S.sh.79%7c4%7c0&IECachePoison=20678851.0608703&Counter5=SS_annotate%7c30092491%7cmesz%7cmedline%7cmedl&Counter5Sessionless=1) |  |
| --- | --- | --- | --- |

|  | 5. | [Changes in Body Mass Index During a 3-Year Elementary School-Based Obesity Prevention Program for American Indian and White Rural Students.](http://ovidsp.dc2.ovid.com.cyber.usask.ca/sp-3.33.0b/ovidweb.cgi?&S=KBLNFPFCGEEBJALKJPDKFGBHJCCIAA00&Complete+Reference=S.sh.79%7c5%7c1&Counter5=SS_view_found_complete%7c28693339%7cmesz%7cmedline%7cmedl&Counter5Data=28693339%7cmesz%7cmedline%7cmedl)  Vogeltanz-Holm N; Holm J.  Health Education & Behavior. 45(2):277-285, 2018 04.  [Journal Article. Research Support, U.S. Gov't, P.H.S.]  UI: 28693339  Authors Full Name Vogeltanz-Holm, Nancy; Holm, Jeffrey.    [My Projects](http://ovidsp.dc2.ovid.com.cyber.usask.ca/sp-3.33.0b/ovidweb.cgi?&S=KBLNFPFCGEEBJALKJPDKFGBHJCCIAA00&FormProcessor=item__add&InputArgs=form_name%3aitem__add%7cinterface_origin%3aoi%7ckeep_selection%3aY%7cdisplay_type%3aTitles%7cdlname%3aS.sh.79%7cstart%3a1%7ccount%3a10%7cindex%3a5%7con_mm_page%3a%7c&Counter5=SS_add_to_my_projects%7c28693339%7cmesz%7cmedline%7cmedl) [Annotate](http://ovidsp.dc2.ovid.com.cyber.usask.ca/sp-3.33.0b/ovidweb.cgi?&S=KBLNFPFCGEEBJALKJPDKFGBHJCCIAA00&Titles=S.sh.79%7c1%7c10&Annotate=S.sh.79%7c5%7c0&IECachePoison=13798866.4691687&Counter5=SS_annotate%7c28693339%7cmesz%7cmedline%7cmedl&Counter5Sessionless=1) |  |
| --- | --- | --- | --- |

|  | 6. | [The experience of gestational diabetes for indigenous Maori women living in rural New Zealand: qualitative research informing the development of decolonising interventions.](http://ovidsp.dc2.ovid.com.cyber.usask.ca/sp-3.33.0b/ovidweb.cgi?&S=KBLNFPFCGEEBJALKJPDKFGBHJCCIAA00&Complete+Reference=S.sh.79%7c6%7c1&Counter5=SS_view_found_complete%7c30518341%7cmesz%7cmedline%7cmedl&Counter5Data=30518341%7cmesz%7cmedline%7cmedl)  Reid J; Anderson A; Cormack D; Reid P; Harwood M.  BMC Pregnancy & Childbirth. 18(1):478, 2018 Dec 05.  [Journal Article]  UI: 30518341  Authors Full Name Reid, Jennifer; Anderson, Anneka; Cormack, Donna; Reid, Papaarangi; Harwood, Matire.    [My Projects](http://ovidsp.dc2.ovid.com.cyber.usask.ca/sp-3.33.0b/ovidweb.cgi?&S=KBLNFPFCGEEBJALKJPDKFGBHJCCIAA00&FormProcessor=item__add&InputArgs=form_name%3aitem__add%7cinterface_origin%3aoi%7ckeep_selection%3aY%7cdisplay_type%3aTitles%7cdlname%3aS.sh.79%7cstart%3a1%7ccount%3a10%7cindex%3a6%7con_mm_page%3a%7c&Counter5=SS_add_to_my_projects%7c30518341%7cmesz%7cmedline%7cmedl) [Annotate](http://ovidsp.dc2.ovid.com.cyber.usask.ca/sp-3.33.0b/ovidweb.cgi?&S=KBLNFPFCGEEBJALKJPDKFGBHJCCIAA00&Titles=S.sh.79%7c1%7c10&Annotate=S.sh.79%7c6%7c0&IECachePoison=83600754.3364282&Counter5=SS_annotate%7c30518341%7cmesz%7cmedline%7cmedl&Counter5Sessionless=1) |  |
| --- | --- | --- | --- |

|  | 7. | [Comparing Indigenous and public health infant feeding recommendations in Peru: opportunities for optimizing intercultural health policies.](http://ovidsp.dc2.ovid.com.cyber.usask.ca/sp-3.33.0b/ovidweb.cgi?&S=KBLNFPFCGEEBJALKJPDKFGBHJCCIAA00&Complete+Reference=S.sh.79%7c7%7c1&Counter5=SS_view_found_complete%7c30458832%7cmesz%7cmedline%7cmedl&Counter5Data=30458832%7cmesz%7cmedline%7cmedl)  Monteban M; Yucra Velasquez V; Yucra Velasquez B.  Journal of Ethnobiology & Ethnomedicine. 14(1):69, 2018 Nov 20.  [Comparative Study. Journal Article]  UI: 30458832  Authors Full Name Monteban, Madalena; Yucra Velasquez, Valeria; Yucra Velasquez, Benedicta.    [My Projects](http://ovidsp.dc2.ovid.com.cyber.usask.ca/sp-3.33.0b/ovidweb.cgi?&S=KBLNFPFCGEEBJALKJPDKFGBHJCCIAA00&FormProcessor=item__add&InputArgs=form_name%3aitem__add%7cinterface_origin%3aoi%7ckeep_selection%3aY%7cdisplay_type%3aTitles%7cdlname%3aS.sh.79%7cstart%3a1%7ccount%3a10%7cindex%3a7%7con_mm_page%3a%7c&Counter5=SS_add_to_my_projects%7c30458832%7cmesz%7cmedline%7cmedl) [Annotate](http://ovidsp.dc2.ovid.com.cyber.usask.ca/sp-3.33.0b/ovidweb.cgi?&S=KBLNFPFCGEEBJALKJPDKFGBHJCCIAA00&Titles=S.sh.79%7c1%7c10&Annotate=S.sh.79%7c7%7c0&IECachePoison=81758048.9704586&Counter5=SS_annotate%7c30458832%7cmesz%7cmedline%7cmedl&Counter5Sessionless=1) |  |
| --- | --- | --- | --- |

|  | 8. | [Stepping Stones to Resiliency following a community-based two-generation Canadian preschool programme.](http://ovidsp.dc2.ovid.com.cyber.usask.ca/sp-3.33.0b/ovidweb.cgi?&S=KBLNFPFCGEEBJALKJPDKFGBHJCCIAA00&Complete+Reference=S.sh.79%7c8%7c1&Counter5=SS_view_found_complete%7c29250869%7cmesz%7cmedline%7cmedl&Counter5Data=29250869%7cmesz%7cmedline%7cmedl)  Ginn CS; Benzies KM; Keown LA; Raffin Bouchal S; Thurston WEB.  Health & Social Care in the Community. 26(3):364-373, 2018 05.  [Journal Article]  UI: 29250869  Authors Full Name Ginn, Carla S; Benzies, Karen M; Keown, Leslie Anne; Raffin Bouchal, Shelley; Thurston, Wilfreda E Billlie.    [My Projects](http://ovidsp.dc2.ovid.com.cyber.usask.ca/sp-3.33.0b/ovidweb.cgi?&S=KBLNFPFCGEEBJALKJPDKFGBHJCCIAA00&FormProcessor=item__add&InputArgs=form_name%3aitem__add%7cinterface_origin%3aoi%7ckeep_selection%3aY%7cdisplay_type%3aTitles%7cdlname%3aS.sh.79%7cstart%3a1%7ccount%3a10%7cindex%3a8%7con_mm_page%3a%7c&Counter5=SS_add_to_my_projects%7c29250869%7cmesz%7cmedline%7cmedl) [Annotate](http://ovidsp.dc2.ovid.com.cyber.usask.ca/sp-3.33.0b/ovidweb.cgi?&S=KBLNFPFCGEEBJALKJPDKFGBHJCCIAA00&Titles=S.sh.79%7c1%7c10&Annotate=S.sh.79%7c8%7c0&IECachePoison=31493726.05492&Counter5=SS_annotate%7c29250869%7cmesz%7cmedline%7cmedl&Counter5Sessionless=1) |  |
| --- | --- | --- | --- |

|  | 9. | [Developing a Tribal Health Sovereignty Model for Obesity Prevention.](http://ovidsp.dc2.ovid.com.cyber.usask.ca/sp-3.33.0b/ovidweb.cgi?&S=KBLNFPFCGEEBJALKJPDKFGBHJCCIAA00&Complete+Reference=S.sh.79%7c9%7c1&Counter5=SS_view_found_complete%7c30581178%7cmesz%7cmedline%7cmedl&Counter5Data=30581178%7cmesz%7cmedline%7cmedl)  Jennings D; Little MM; Johnson-Jennings M.  Progress in Community Health Partnerships. 12(3):353-362, 2018.  [Journal Article]  UI: 30581178  Authors Full Name Jennings, Derek; Little, Meg M; Johnson-Jennings, Michelle.    [My Projects](http://ovidsp.dc2.ovid.com.cyber.usask.ca/sp-3.33.0b/ovidweb.cgi?&S=KBLNFPFCGEEBJALKJPDKFGBHJCCIAA00&FormProcessor=item__add&InputArgs=form_name%3aitem__add%7cinterface_origin%3aoi%7ckeep_selection%3aY%7cdisplay_type%3aTitles%7cdlname%3aS.sh.79%7cstart%3a1%7ccount%3a10%7cindex%3a9%7con_mm_page%3a%7c&Counter5=SS_add_to_my_projects%7c30581178%7cmesz%7cmedline%7cmedl) [Annotate](http://ovidsp.dc2.ovid.com.cyber.usask.ca/sp-3.33.0b/ovidweb.cgi?&S=KBLNFPFCGEEBJALKJPDKFGBHJCCIAA00&Titles=S.sh.79%7c1%7c10&Annotate=S.sh.79%7c9%7c0&IECachePoison=63128423.8378154&Counter5=SS_annotate%7c30581178%7cmesz%7cmedline%7cmedl&Counter5Sessionless=1) |  |
| --- | --- | --- | --- |

|  | 10. | [The cost-effectiveness of a 20% price discount on fruit, vegetables, diet drinks and water, trialled in remote Australia to improve Indigenous health.](http://ovidsp.dc2.ovid.com.cyber.usask.ca/sp-3.33.0b/ovidweb.cgi?&S=KBLNFPFCGEEBJALKJPDKFGBHJCCIAA00&Complete+Reference=S.sh.79%7c10%7c1&Counter5=SS_view_found_complete%7c30260984%7cmesz%7cmedline%7cmedl&Counter5Data=30260984%7cmesz%7cmedline%7cmedl)  Magnus A; Cobiac L; Brimblecombe J; Chatfield M; Gunther A; Ferguson M; Moodie M.  PLoS ONE [Electronic Resource]. 13(9):e0204005, 2018.  [Journal Article. Randomized Controlled Trial. Research Support, Non-U.S. Gov't]  UI: 30260984  Authors Full Name Magnus, Anne; Cobiac, Linda; Brimblecombe, Julie; Chatfield, Mark; Gunther, Anthony; Ferguson, Megan; Moodie, Marj.    [My Projects](http://ovidsp.dc2.ovid.com.cyber.usask.ca/sp-3.33.0b/ovidweb.cgi?&S=KBLNFPFCGEEBJALKJPDKFGBHJCCIAA00&FormProcessor=item__add&InputArgs=form_name%3aitem__add%7cinterface_origin%3aoi%7ckeep_selection%3aY%7cdisplay_type%3aTitles%7cdlname%3aS.sh.79%7cstart%3a1%7ccount%3a10%7cindex%3a10%7con_mm_page%3a%7c&Counter5=SS_add_to_my_projects%7c30260984%7cmesz%7cmedline%7cmedl) [Annotate](http://ovidsp.dc2.ovid.com.cyber.usask.ca/sp-3.33.0b/ovidweb.cgi?&S=KBLNFPFCGEEBJALKJPDKFGBHJCCIAA00&Titles=S.sh.79%7c1%7c10&Annotate=S.sh.79%7c10%7c0&IECachePoison=2166736.64691527&Counter5=SS_annotate%7c30260984%7cmesz%7cmedline%7cmedl&Counter5Sessionless=1) |  |
| --- | --- | --- | --- |

All

- [About UsOpens in new window](http://www.ovid.com.cyber.usask.ca/site/about/index.jsp?top=42)
- [Contact UsOpens in new window](http://www.ovid.com.cyber.usask.ca/site/support/techSupportForm.jsp?top=34&mid=35)
- [Privacy PolicyOpens in new window](http://ovid.com.cyber.usask.ca/site/privacy.jsp)
- [Terms of UseOpens in new window](http://www.ovid.com.cyber.usask.ca/site/about/terms.jsp)

© 2019 [Ovid Technologies, Inc.Opens in a new window](http://www.ovid.com.cyber.usask.ca/) All rights reserved.

OvidUI_04.01.00.001, SourceID 118528
