# Supplementary material for: Factors of success, barriers, and the role of frontline workers in Indigenous maternal-child health programs: a scoping review
Source: Int J Equity Health. 2024 Feb 13;23:28. doi: 10.1186/s12939-024-02118-2 (PMC10863162; doi:10.1186/s12939-024-02118-2)
Supplement: Supplementary file 2 — Additional file 2. [file 12939_2024_2118_MOESM2_ESM.docx]

| Author, Year,  Title | Indigenous  First Author | Country  Pop. | Meth. | Aims/  Purpose | Program  Description | Reported Factors of Program Success | Reported Program Barriers | Frontline Worker  Role in Health Program  Process | Program  Outcomes |
| --- | --- | --- | --- | --- | --- | --- | --- | --- | --- |
| Abbott et al., 2013  *Improving immunisation timeliness in Aboriginal*  *children through personalised calendars* | Not stated in bio  First author works for AMSWS | AUS  Aboriginal and Torres Strait Islander children 0-2 years  and carers | Quant - Pre and post measures of effectiveness  N=505 | -To prompt timely immunization and increase rates | - Carers offered calendar at immunization appointment  - Calendars included picture of child, Aboriginal artwork, health promotion messages | - Photo and artwork identified as most valued feature  - Enhanced clinical interaction with clients  - Expression of self-identity  - Low cost, easy to implement  - Little impact on service delivery  - Participants felt celebrated and expressed their cultural identity  - Staff members felt helped to prompt immunization | - Staff shortages  - Clinic space  - High client volume  - Technology challenges (computer not always available)  - Staff unsure of value of program  - Competing demands  - Carers felt unable to ask for calendar  - Long clinic wait times | - Practice nurses and AHWs delivered the program  - Contributed to the evaluation  - Consumers of program included in program development through a survey  - No evidence of FLWs in program the development | - Effective to prompt next immunization  - Increased timeliness of immunizations  - Educational effect on parents  - Carers reported helped remind them to vaccinate  - Timeliness of immunizations increased afterwards |
| Arnold et al., 2011  *The Coming of the Blessing*  *A Successful Cross-Cultural Collaborative*  *Effort for American Indian/Alaska*  *Native Families* | Not stated in bio  Community members in list of authors | US  AI/AN  Women of child-bearing years | Case study  Survey to assess culturally appropriateness of resource  7500 copies distributed, 181 survey responses from mothers | - Community members identified need to support young pregnant women in traditional manner  - Created a Prenatal education program created and taught by community women | - Booklet is an education tool from conception to after the baby is born  - Includes info on pregnancy, breastfeeding, immunization | -Trust within committee  Protocols such a prayer, gift giving  -Community members took the lead and felt “empowered”  - “Culturally appropriate” by basing the program on the Medicine Wheel, AI/AN protective factor, inclusion of baby’s father  - AI/AN artist contributed to booklet | -No areas of improvement reported | - AI/AN committee women represented both lay and professional health care providers  - Role in development and evaluation of the program  - FLWs delivered the booklet and program | - 88% found booklet helpful - 77% started prenatal care. -88% kept all prenatal appointments  - Pre-term birth rate 7% versus 14.1% in all AI/AN births  - Reported behaviour changes such as eating better, reducing stress, exercising and deciding to breastfeed |
| Balmer & Foster,  1997  *Preliminary evaluation of the effects of a*  *nutrition awareness project on the*  *Ngaanyatjarra Pitjantjatjara Yankunytjatjara (NPY)*  *communities of central Australia* | Not stated in bio.  Second author is Indigenous and article endorsed by NPY women’s council. | AUS  Aboriginal women and children  Young mothers and children under 5 | Process Evaluation quant- descriptive stats; narrative  Outcome Evaluation  Anecdotal evidence  Impact Evaluation  Quant  N= 8 communities, 212 mothers and children | - Nutrition project  - Develop awareness and knowledge to prevent failure to thrive  - Promote health practices.  - Maintain better health  - Project initiated by the  NPY Women's Council | - Outreach and education  - Crisis intervention  - Production of a culturally appropriate resource manual  - Store education and recommendations | - In community language  - Senior community women promoting program  - Full support of community health clinic  - Culturally appropriate for community.  - Aboriginal leading workshop  - Initiated by NPY Women’s Council | - Attendance decreased over lessons  - Community obligations and demands of mothers made participation challenging  - Gender roles of women have created challenges  - Not able to engage fathers in program | - Indigenous senior health worker, a non-Indigenous registered nurse, and a play therapist delivering program  - Indigenous health worker in training to take over play therapist role  - Role of the workers in planning and evaluation not explicitly identified ?may have contributed to evaluation component | - Educational component has been effective  - Certain problem areas identified in crisis intervention  - Challenging to influence stores  - No reduction in junk food consumption  - No change in hospital admissions  - Some children gaining weight  - Identified need for intensive and comprehensive interventional approach  - Challenging to determine program effect as mothers reluctant to speak out in evaluation |
| Banks, 2003  *Ka’nistenhsera*  *a Native community rekindles the tradition of*  *breastfeeding* | Unknown  Cannot find a bio | CAN  Quebec  First Nations  Community  Mohawk | Community-based  Intervention  Driven by community  Quant – descriptive stats, BF rates and changes  No N reported, just timeframe of program,  1995-2001 | - Increase BF rates in initiation and sustained BF  - Promote normalcy of BF through a core groups of BF moms  - Raise community awareness about B | - Trained a community member (grandmother) as a BF promoter  - Provided pre and postnatal support  - Initiated a mother’s support group  - Articles published in community newspaper  - Education in school on BF  - Talking Circle with Elders to promote BF | - Culturally-based program  - Strengths-based  - Included strong kinship  - Deep respect for Elders  - Connection with natural world  - Grounded in cultural competency  - Multi-faceted  -Community participation and empowerment  - Oral traditions for education component  - Collaboration with the community as a whole (includes other health professionals)  - Community member as BF promoter identified as most effective tool in regenerating BF  - Uniqueness of each community imperative for success  - Preferred peer experiential learning | - Formal presentations to the mother’s group not well received  - Conventional written education materials not well received  - Family members wanting to participate in feeding  - Community beliefs and feeding practices  - Formula provided free to women on welfare  - Grandmothers’ beliefs could best help new mothers by letting them rest | - Program development included the community health workers and community nurses’ input  - Community member delivered program | - Improvement in initiation and duration of breastfeeding rates  - Increases of 43% and 23% from pre-intervention  - Other women in the community willing to come forward and provide BF support  -Unable to establish core group of BF women |
| Barlow et al., 2018  *Discovering frugal innovations through delivering early childhood home visiting interventions in low-resource Tribal communities* | Not stated in bio  Second author McDaniel is Choctaw Nation of Oklahoma. Article was reviewed and approved by Choctaw Nation of Oklahoma, Native American Health Center, Kodiak Area Native Association, and White Mountain Apache Tribe | US  AI/AN | Narrative of program barriers and solutions.  No N reported | - To foster positive parenting, healthy child development and improve family wellness  - Home-visiting in low-income and hard-to-reach AI/AN communities | - Culturally appropriate home visiting program  - Includes education, referrals and support | - Prioritized relationship and trust building  - Local Indigenous home visitor fluent in local language.  - Flexibility of program for cultural enhancement, meet families where they are at  - Can take a break from program if need to  - Not a defined inclusion criteria  - Elastic home-visiting, home visits took place anywhere, with flexible times  - Role of Elders  - Included extended-family networks  - Persistence in recruiting and retaining families  - Alternate program delivery for bad weather  - Developing safety protocols and staff support plans | - Lack of community trust  - Concern will not last due to funding, - Historical “do to” community  - Transportation for families and home visitors (roads)  - Weather challenges  - Safety of home visitors and participants (domestic violence, dogs, active crime)  - Risk of high turnover of home visitors  - Inadequate resources and capacity to manage data and evaluation.  - Negative perception of assessments  - Fractured service networks – long wait times, geographic isolation  - Requirements of funding program | - Delivered by an Indigenous paraprofessional from the community  - FLWs engaged in quality improvement and central role in community wellness. ? role in development and evaluation | - No evaluation component or discussion of outcomes  - All communities agreed that programs legislatively funded and locally run addressed disparities in a culturally appropriate manner |
| Bertilone and McEvoy,  2015  *Success in closing the gap: Favourable neonatal*  *outcomes in a metropolitan Aboriginal Maternity Group Practice Program* | Unable to find bio | AUS  Indigenous pregnant women  in south metropolitan Perth | Non-randomized intervention with 2 control groups  Neonatal health outcomes measured.  Qual  program evaluation  N=343 | - To improve access to existing antenatal and maternity services  - Increase births in local hospital | - Indigenous grandmother, Aboriginal Health Officers and midwives delivered standard antenatal visits  - Outreach services included health education, health promotion  - Home visit and clinic services provided | - Driven by Aboriginal community members  - Collaborative with program staff and health unit staff  - Grandmothers respected in local community with good community networks  - Partnership with local care providers  - Program model culturally secure  - Culturally appropriate, holistic interventions  - Transportation provided | - Selection bias of program, different risk profiles of program participants  - No program barriers or areas for improvement reported within the article | - Grandmothers and Aboriginal Health Officers delivered the program  - Input in planning  - Participated in qualitative interviews to assess program | - Sig. improved neonatal health outcomes  - Less preterm births (9.1% vs 15.9% and 15.3%); resuscitation (17.8% vs 24.4% and 31.2%); hospital stay greater than 5 days (4.0% vs 11.3% and 11.6%)  - Participation in program continued to grow.  - More local births  - Improved antenatal care uptake among Aboriginal teenagers |
| Bertilone et al., 2017  *Elements of cultural competence in an Australian Aborigianl maternity program*  Goes with  Bertlilone and McEvoy, 2015 | Unable to find bio  3^rd^ and 4^th^ authors are Aboriginal (Gower and Naylor) | AUS  Aboriginal pregnant women in south metropolitan Perth | Qual  Evaluation  n=16 program participant; n=22 from partner agencies;  n=15 staff interviews  N=53  Aboriginal participant  n= 7 staff;  n=16 clients;  n=8 from partners organizations | - Focused on program staff, hospital, and community health service providers  - To explore the elements of the program that contributed to cultural competence | As in previous article Bertilone & McEvoy, 2015 | - Driven, tailored to community  - Community governance  - Aboriginal staff  - Fostered relationship between clients and other agencies  - 2-way learning process between Aboriginal and non-Aboriginal staff  - Yarning  - Grandmothers (Elders) could influence clients with their respected position in community  - Continuity of care model engaged clients long-term  - Partnerships essential to address social determinants of health  - Supportive leadership and management  - Transportation provided  - Home visits essential to improve access and build trust | - Working with existing services posed challenges when infrastructure and policies did not support Aboriginal family practices  - Barrier to culturally competent service  - No standard mechanisms to measure client satisfaction  - Expectations on midwives to use their time to see non-AGMPP patients | - Steering committee (community members and partners) guided the evaluation design  - Staff were aware of evaluation purpose, approached to participate, but do not appear to be a part of the design | - Supportive leadership and management had the most evidence for improvements in culturally appropriate care  - Advocacy from program staff resulted in some policy change  - Partnership resulted in more culturally appropriate care  - Continued beyond study funding, embedded as a standard practice  -Ongoing employment of staff  - Cultural appropriateness of care improved  - Reconciliation Action Plan developed and implemented  - Clients will access other services, feel more comfortable |
| Bowes & Grace, 2014  *Review of early childhood parenting education and health intervention programs for Indigenous children and families in Australia* | Not stated Bio  talks about “walking alongside Indigenous Peoples” | AUS  CAN  US  NZ  Indigenous children  From conception to school entry (age 5 or 6) | - Review of peer-reviewed and grey literature | - Review of prevention and early intervention research literature to improve outcomes for Australian Indigenous children | - Reported on a variety of parenting, early childhood health and education programs | - Strengths-based  - Family-centred approach  - Flexibility  - Sustainability  - Adaptations to the local context, culture, needs, and language  - Models of service integration and collaboration  - Community support  - Building trust and establishing relationships  -Employ local community members  - Program run in places that are perceived as safe where participants feel a sense of ownership and control  - Appropriate training and support  - Cultural safety  - Choice of non-Indigenous staff crucial  - Account for historical context | - Disrespectful manner, sends negative messages about Indigenous people’s knowledge  - Cannot be approached same as mainstream programming  - Assumption communities are the same  - Lack of evaluation | -Should participate and guide the delivery of the program (Nurses, Indigenous community workers) | -Various program outcomes: positive children’s outcomes, stronger parenting skills, increased knowledge surrounding child health |
| Campbell et al., 2018  *Implementing the Baby One program*  *: A qualitative evaluation of family-centred child health promotion in remote Australian Aboriginal communities* | Unable to find bio  1^st^, 4^th^, 5^th^, 6^th^, and 7^th^ authors employed by Apunipima Cape York Health Council | AUS  Cape York Indigenous communities in Queensland | Qual evaluation  Using semi-structured interviews and a focus group  N=48  24 Apunipima staff members (Indigenous Health- workers, Men’s Healthworkers, other health practitioners (nurses, midwives, managers, admin)  4 family members in program  20 men in focus group | -To improve long-term health and give children the best start to life  - To determine how the program was implemented, enablers, and formative implementation outcomes  N=161 pregnant women and families in program | - Structured IHW-led family visiting program  - Begins at pregnancy and continues until the child reaches 2yrs10mos  - Includes 7 baby baskets and relevant health promotion “yarning” topics and activities  - Developed by Apunipima Cape York Health Council | -Relationships with families, respect  - Positive language  - Family-centered, family-led, whole family  - Opportunity for knowledge exchange between Indigenous Healthworkers and other providers  - Indigenous workforce builds culturally-safe relationships with community, connect to resources  - Communication style is important (no jargon)  - Home visits  - Yarning  - Consistent worker  - Supporting and training IHWs  - Indigenous leadership  - Including Indigenous knowledge/practices  - Values and respects local cultural beliefs and practices | - Exclusion of fathers  - Overcrowding of households  - High staff workload  - Long distances and high costs impede formal training and bringing staff together  - Need more resources allocated  - Focus on health workers meant that some team members felt excluded  - Health workers may face cultural and kinship barriers  - Challenging home environments and lack of family support  - Division between Health workers and nurses/midwives  - Role confusion | -FLWs delivered the program and program development | - Good health through behaviour change (i.e., quitting smoking and alcohol)  - Reduced risk of families engaging with child welfare  -More comfortable to access local clinic and engage with staff  - Need formal ongoing evaluation developed in a collaborative process |
| d’Espaignet et al., 2003  *Monitoring the ‘Strong Women, Strong Babies, Strong Culture Program The first eight years* | Unable to find bio | AUS  Aboriginal women and infants | Quant  Linear regression  Comparison of pre and post birth wt in intervention and control communities | - To assess the improvements in birth wt and changes in additional intervention groups  - To increase birth wts and reduce Aboriginal infant mortality rates | - For senior women in Aboriginal communities to support younger Aboriginal women in pregnancy | - Senior Aboriginal women to deliver the program  - Culturally appropriate and attuned care and education  - Fluid program of individual and community support  - Tailored to personal, social circumstance of pregnant women, services available in community, skill level of workers  - Community empowerment, self-determination | - Contents of program different b/n 2 groups  - High health and welfare needs of Aboriginal people  - Low resource levels  - Need better coordinated services  - Computerized information systems to link service providers  - Less support of workers in some intervention communities | - Local women delivered program  - Program development and eval design not discussed | - Sig. improvements in birthweight for Group 1 communities  - Maintained over time with no sig change Group 2  - Wider social and economic impact of program with opportunities for gainful employment, earnings, and Aboriginal people tackling own issues  - Need in-depth qual methods to identify strengths and weaknesses of program  - Not clear what impact the SWSBSC program had on specific aspects of antenatal care |
| Duffy et al., 1994  *Community Baby Shower as a Transcultural Nursing Intervention* | Not identified in bio | US  AI  Southeastern Michigan  Adolescent mothers pregnant women and women with newborn up to 6 months | Case study  Pre/Post intervention oral knowledge assessment  No N reported | - To determine the health education needs of Native American women whose infants were at risk for infant mortality  - To educate mothers about newborn care, immunizations and infant safety | -Community baby shower with a health education focus  - Videos, informal discussion, written information, games, and prizes | - Invited friends and relatives of participants  - Peer group activity  - Culturally based  - Flexible schedule  - Traditional spiritual leader from the community  - Native American imagery theme  - Students’ relationships with organizations enhanced likelihood of donations  - Word of mouth, flyers, posters for advertising  - Community needs assessment conducted  - Atmosphere of positive feedback and idea exchange  - Multiple human and community resources included | - Fund raising and locating items for the shower was challenging  - Time and resource constraints  - Need accurate inclusion criteria in advertising to avoid turning people away | -Nursing students were involved in the development, planning, implementation, and evaluation of the intervention | - Oral knowledge tests and demonstrations of  baby care skills completed successfully by all participants  - Positive community response to baby shower  - Self-confidence of mothers increased after attending baby shower |
| Eni et al., 2009  *Trend setting in ManitobaThe challenge of designing the First Nations Maternal Child Health Strengthening Families Program in Manitoba* | Yes  Fox Lake Cree Nation in northern Manitoba | CAN  Manitoba  First Nations families on-reserve | Discussion Paper | - To promote strong, healthy and supportive First Nations families  - Reduce health and service disparities between First Nation communities and non-Aboriginal communities  - Increase participation rates in prenatal care services | -First Nations home visiting program (federally funded).  Manitoba Child Health Strengthening Families (MCH-SF)  -Mainstream and traditional Aboriginal health care approaches | - Responsive to cultural and contextual appropriateness  - First Nations driven.  - Supportive of self-governance  - Formulated in a collaborative context.  - Involves voices of families  - Principles of self-determination  - Not a static program, but continually evolving  - Partnerships with Elders and other community-based programs | - Balancing mainstream program design and Aboriginal cultural integrity  - Limited First Nations specific research to draw on for program design | -Public health nurses and paraprofessionals deliver support, advocacy, and education to childbearing women, new mothers and families | - Not reported  - Identified issues to be assessed: impact of delivering program activities and developing activities on First Nations health  - Understanding of principles for meaningful collaboration between agencies and incorporate community families |
| Gerlach & Gignac, 2019  *Family engagement and well-being in Aboriginal Head Start Programs in Canada a Qualitative Inquiry* | No  2^nd^ author is employed by Aboriginal Head Start Association of BC – did not participate in data collection or analysis | CAN  B.C.  12 programs across B.C. serving approx. 755 children and families | Qual  CBPR  Semi-structured interviews  N=26 participants  10 -parents  10- AHSUNC coordinators and family workers  6-Elders | - Generate knowledge and insights into family engagement in AHSUNC programs  - For families to get maximum benefits out of the early childhood program by being actively engaged  - To mitigate the impact of early adversity on children | - AHSUNC federally funded, developed by Indigenous community organization - Operates from Sept – June, half day preschool for children aged 3-5 | - Prioritized time to build trust, relationship with parents  - Community relationships and networks  - Tailor engagement and strategies  - Strengths-based approaches  - Supporting the whole family, embracing a broader scope of practice  - Co-locating with other Indigenous community agencies or hubs  - Having community governance  - Indigenous cultural knowledge/practices  - Flexible and responsive  - Using Facebook  - Elder involvement  - Transportation  - Parent friendly space in program, welcoming | - Mental health of parents impacts capacity to engage in program  -Program mandated by child services creates barrier to engage family  - Fear of having children removed  - Multiple stressors for families – food insecurity, mental health, substance abuse  - Engage with more fathers and male caregivers  - Families’ histories and experiences with residential school, child welfare can impact engagement and program | - Deliver the program  - Participated in interviews for study (Evaluation)  - No commentary on program development or evaluation | - Continuity between relational practices that work to foster family engagement and family well-being  - Relational approaches are beneficial to engage all families, but are extremely relevant when attempting to engage with families experiencing stressors and social disadvantages  - Study results were consistent with lit and other studies on Indigenous early childhood programming |
| Gerlach et al., 2017  *Engaging Indigenous families in a community-based*  *Indigenous*  *early childhood programme in British Columbia, Canada:*  *A cultural safety perspective* | No | CAN  B.C.  Indigenous children 0-6 yrs and families in off-reserve urban settings | Qual  Semi-structured interviews  N=35  10 -  Indigenous primary caregivers  4- Elders  18 AIDP workers  3- administrative leaders | -To examine and  analyse how AIDP workers supported family and children’s  health and well-being  -To increase the number of Aboriginal families engaging in early childhood programmes | - AIDP Program unique to B.C.  - AIDP workers provide home visiting, outreach, and group programs | - Relationships and trust with participants essential  - Taking the time to build the relationship  - Understanding historical intergenerational and current concerns surrounding child welfare system  - Workers reflecting on place of privilege to examine impact on work as part of providing culturally safe care  - Elder participation.  - Indigenous knowledge and practices  - Flexible model of program delivery with client driving  - Supporting the whole family, centering their priorities  - Voluntariness of program  - Adapting program and policies to families | - Mistrust, historical and/or current involvement with child welfare  - Organizational and structural challenges to give  primacy to relationships, time required was misunderstood, undervalued, or dismissed by leadership and colleagues  -Time for relationships not captured or recognized in reportable stats to ministry  - Structural policies and practices such as paper work can be a barrier to engagement of clients  - Broadening the scope of the workers, does raise education and training concerns | - Delivery of program  - No evidence or discussion of program development or evaluation | - Not specifically identified or measured in article  - Identified how AIDP was transforming early childhood policies and practices to be responsive to Indigenous families and engage them in early childhood program |
| Glover et al., 2016  *Could ‘Aunties’ Recruit Pregnant Indigenous Women Who*  *Smoke Into a Trial and Deliver a Cessation Intervention?*  *A Feasibility Study* | Not stated.  3^rd^ author may be Indigenous – Te Poutokomanawa | NZ  Ringa Atawhai  Maori Aunties and pregnant Maori women who smoked | Feasibility study  PAR  Mixed Methods  Survey Face-to-face interviews n=14 mothers;  n=5 Aunties  Hospital birth records examined  n=67pregnant women who smoked  88%  Maori  n=8  Aunties | -Test the feasibility of an acceptable smoking cessation intervention  - To reduce smoking while pregnant and protect the fetus from risk  - To increase early registration with a Lead Maternity Carer | - Aunties advised the pregnant women to abstain from smoking, offered subsidized nicotine replacement, or referral to local cessation providers  - A booklet on healthy eating for pregnancy was provided and help to register with a Lead maternity Carer (LMC) | - Participants described Aunties as supportive, nice, non-judgmental used principles of love, compassion, and support  - Aunties know and are involved in their community  - Could relate and interact with participants in a culturally appropriate way  - Work at grass roots level, use spiritual connectedness and other Maori paradigms  - Able to give a gift pack  - Community involvement in all aspects of study  - Auntiese worked in Maori way with principles and practices | -Lack of follow-up by Aunties  - Women had over-riding issues (gang lifestyle, abused)  - Needed trained person employed for the project to refer moms  - Should include the wider family, social circle  - Aunties needed more training.  - Lack of continuity of Aunties challenging (reality of relying on volunteers)  - Completing detailed logs did not work  - Aunties did not attend all the training and could not deliver all aspects of the program | - Aunties delivered the intervention, participated in the evaluation  - No discussion on how the Quit Card was developed or what to put in the gift basket | - Aunties were able to deliver an acceptable intervention  - Identified feasibility issues related to intervention delivery and data collection |
| Harrison et al., 2010  *Design and implementation of a dental caries*  *prevention trial in remote Canadian Aboriginal*  *communities* | Not stated  2^nd^ author employed by Cree Board of Health and Social Service of James Bay | CAN  Quebec  James Bay  Aboriginal (Cree) mothers and children 0-30 months in age | RCT  By community  9 Cree community  5 – intervention group  4-control groups  N=272 mothers | - To test the effectiveness of a counseling approach (MI) to control dental caries in Aboriginal children  - To reduce dental caries in the test communities by 20%  - To decrease numbers of children requiring anesthesia or sedation for dental treatment | - Aboriginal women recruited and delivered counselling to expectant and new mothers in the test communities  - MI session during pregnancy, several more up to child 2yrs  - Received dental health resources (toothbrushes, paste, sippy cup, FV) | - MI fits well with the Cree philosophy (suggesting vs directing)  - Resources provided  - Culturally appropriate educational pamphlet  - Early and continuous community collaboration  - Sensitive to existing beliefs, traditions about parenting practices.  - Local workers deliver the intervention  - Sought staff engagement (buy-in) and provided training  - Project manager from community, maintained regular contact with staff | - Community consultation complex, 2 years of advance planning  - Initial community concerns about randomization process  - Trial location in remote northern Canada  - CHRs felt overworked and not sufficiently rewarded for addition to daily workload  - Recruitment challenges, mothers too busy or not interested in participating  - Clarifying who would do the day-to-day work for the trial – community wanted existing Health Department staff rather than new staff ultimately split the project tasks between existing staff and new staff | - Delivered by local CHRs  - Health care workers part of 2-year consultation process with community to develop project, strategies  - CHRs engaged in development of scripts, menus for intervention | - Identified challenges and successes of research project process  - Established baseline for mothers at beginning of program |
| Health Council of Canada, 2011  *Understanding and Improving Aboriginal Maternal and Child Health in Canada*  *Conversations about Promising Practices across Canada* | No | CAN  First Nations, Inuit, and Metis expectant mothers and children from prenatal to age 6 | Qual  Regional meetings of frontline workers, program managers, academic, gov’t reps  from urban and rural, northern and  southern settings.  First Nations,  Inuit and Métis communities were brought together | -To determine what is working from people in the field |  | - Holistic view of health, family and community context  - Strong relationships, mutual understanding, respect  - Collaborating, combining funding, programs, services  - Culture – language, traditions, self-determination  - Integrating traditional approaches in health programs  - Community-based  - Developed or adapted to the local level  - Training and human resources  - Aboriginal workers.  Rebuilding pride and Aboriginal language, traditions, culture, self-determination  - Cultural sensitivity and safety in care, delivery and training  - Multi-disciplinary team | - Clash of values  - Impact of colonialism, trauma, residential schools  - Non-Aboriginal providers not understanding or valuing multi-generational effects or Aboriginal ways  - Paternalistic tx  - Racism  - Fear accessing programs will result in child welfare involvement  - Funding, shortage of stable, long-term funding, not meeting program demand, complex applications and structure  - Staff burnout  - Distance, transport costs  - Jurisdictional issues b/n fed, prov, municipal, health authorities, band councils | -Program delivery | -Practices deemed effective and successful from the perspective of the participants  - Identified determinants of health (living conditions and circumstances) that impact Aboriginal mothers and children  - Identified the key barrier to good programs is the lack of stable, multi-year funding or lack of enough funding to meet needs of population size |
| Health Council of Canada, 2013  *Understanding and improving Aboriginal maternal and child health in Canada*  *Compendium of promising practices* | No | CAN | Compendium of “promising practices”  Good definition of promising practices on page 1 | -Compilation of promising practices that goes with Health Council of Canada, 2011 | -There is a need to share practices amongst stakeholders (federal, provincial and territorial to frontline community staff) | - Traditional knowledge and cultural approaches -language, holistic approaches  - Community-based and community-focused approaches  - Collaborative and integration - bringing together, working together, combining funding, linking with other community programs and services  - Training and human resources - recruiting, training, and retaining Aboriginal people, (cultural competency, cultural safety)  - Policy and funding -broad policies or funding at the federal, provincial, territorial or Aboriginal gov’t level that have improved maternal and child health | - Not addressed in document | - Participated in the offered sessions to contribute knowledge on program successes and barriers | - Identified 5 key themes of promising practices. incorporated  - Created document that listed all the promising practices from more than 100 programs |
| Hiratsuka et al., 2018  *Cultural adaptations of evidence-based home visitation models in tribal communities.* | Yes | US  AI/AN  Families  Pregnant to 2yrs of age | Qual  CBPR  Case Studies of the development of 4 Tribal MIECHV  programs | - Describe how Tribal MIECHV program assessed community needs, selected a home visiting model and the cultural adaptation process | - To implement evidence-based home-visiting services for AI/AN families  - Visits provided by lay health workers, registered nurses, or AI/AN paraprofessional | - Surface Cultural Adaptations:  AI/AN imagery in program and recruitment materials (photos, art work)  -Deep Structure Cultural Adaptations:  Curriculum content based on cultural ways, traditional parenting  -Elders involved in program, extended family  - AI/AN home visitors  - Community partnerships  - Using AI/AN languages and values  - Group activities  - Support to address determinants of health, referrals  - Staff training -community input into staff training  - Indigenous perspective must ground all processes | - Historical trauma  - Loss of languages  - Racism and discrimination  - Service providers involving Child Protective Services on the basis of race versus risk to children  - Structural Inequities- determinants of health, poverty, housing  - Transportation  - Only families meeting high risk offered program  - Not all programs have all AI/AN staff  - Large, remote geographical area | - Frontline workers contributed to the development of the program - input through interviews and focus groups  - One program got feedback from staff to inform evaluations | -Not identified in article; identified as area for more research  - Did community need assessments  - Study had limited ability to assess effectiveness and implementation of culturally adapted home-visiting interventions |
| Lawrence et al., 2004  *Effects of a community-based prenatal nutrition program on the oral health of Aboriginal preschool children in Norther Ontario* | Not stated in bio  2^nd^, 3^rd^, 4^th^, and 5^th^ authors are employed by Sioux Lookout Zone Dental Program | CAN  Ontario  Sioux Lookout  Ojibway and Cree prenatal women and children up to 12 months postpartum | Quant  Cross sectional and longitudinal  N=705 caregiver and child pairs | -To improve the nutritional status and well-being of prenatal women and children, promote oral health  - Assess the effectiveness on parents beliefs and behaviours of dental preventive practices and feeding habits of infants and toddlers | -Delivered by a nutrition educator through home visits to provide culturally appropriate community-specific nutrition and dental preventive education | - Informational pkg written in both English and Oji-Cree  - Broad-based program that focus on both child and caregiver  - Support of program by community health workers  - Community-based program  - Ownership by community enhances success and sustainability  - Use of visual aids  - Involvement of Aboriginal peoples in design and implementation of community-based intervention program  - Home visits are one-on-one, culturally appropriate, and community specific  - Program activities and promotion are multi-agency and collaborative | -Determinants of health create strain that lead to poor oral hygiene and dietary habits  - Geographical barriers that make access to dental care difficult | - Deliver the program to clients (community dental assistants)  - No discussion on input in program development or evaluation | -Sig positive impact on caregiver’s knowledge, beliefs and attitudes in relation to infant’s oral health  - Caregivers reported cleaning child’s teeth more frequently and at an earlier age  - More children seen by a dental professional  - Increased dental tx under general, crown, and missing teeth increased with age regardless of program  - Dental caries experience remained very high – over 90% of children were classified as cases |
| Lawrence et al., 2008  *A 2-year community-randomized controlled trial of fluoride varnish to prevent early childhood caries in Aboriginal children* | Not stated in bio  2^nd^ author is employed by Sioux Lookout Zone Dental Program, FNIHB | CAN  Northern Ontario  Sioux Lookout  Zone | Community RCT  Developed with community  N= 20 communities;  1275 children aged 6 months to 5 yrs  Results compared with non-Aboriginal children | - To measure the effectiveness of fluoride varnish and caregiver counselling in preventing ECC  - To prevent ECC, reduce caries increment, and oral health inequalities  - To reduce number of young children receiving GA dental treatment for ECC | -Intervention group received FV 2times per yr with caregiver counselling  - Control group received counselling and usual dental procedures | - Community support of program resulted in high participation rates  - Dental hygienists and program travelled into communities  - FV applications linked to larger mother and child nutrition community-based program | - Parents mobile or difficult to reach  - Parents and children not showing up for appointments  - Children removed to foster care, sick, deceased, or uncooperative during exams  - Children removed from study due to losing all remaining teeth or allergic reactions to FV  - Effect of clustering on the trial results | - Dental hygienist examiners delivered the program. Does not appear to be a community member  - Community dental assistants translated during counseling and assisted during examinations  - Staff recruited parents and obtained consent  - No discussion of development or evaluation design | - Reduced the levels of ECC among a high-risk population  - Fluoride varnish was effective and a safe mode of delivery  - FV reduced the 2-year mean ‘net’ dmfs by 18% for Aboriginal children and 25% for all children  - FV was a very safe mode of fluoride deliver for young children  - Evidence supports shifting some resources towards preventative care and health promotion strategies |
| Lowell et al., 2015  *Supporting Aboriginal knowledge and practice in health care: lessons from a qualitative evaluation of the* *strong women, strong babies, strong culture program* | Not stated in bio  4 Aboriginal Program Coordinators participated as advisors to study | AUS  Northern Territory  Aboriginal mothers and babies | Qual  evaluation  Semi-structured interviews  N=76  6 program coodinators  15 Department of health Staff  15 Strong Women Workers  40 Staff of local organizations and community members | -To evaluate enabling factors and barriers to successful program implementation and future program development  - To evaluate the role of Aboriginal cultural knowledge and practice within the SWSBSC program  - To improve the health of Aboriginal women and their babies; prevent and promote early intervention of lifestyle diseases; strengthen family unit and bring back cultural practices | -SWSBSC initiated in 1993, still operating today  - Employs Strong Women Workers from community to provide support, cultural, and mainstream health education | -Traditional health knowledge and practices essential (ceremonies, traditional medicines)  - Flexibility to adapt to specific community needs  - Traditional parenting, lifestyle, ceremonies  - Strong community development focus  - Cultural events  - Sharing knowledge b/n cultures, i.e., Aboriginal and non-Aboriginal staff  - Effective partnerships  - Employment of Aboriginal women as Program Coordinators to support the workers  - Community control of program crucial for participation  - Committed staff at all levels | - Cultural features of program may have diminished with a shift to mainstream health knowledge and practice  - Lack of respect and recognition of Aboriginal knowledge and practice by some health staff  - Transportation  - High staff turnover and lack of cultural competence  - Power imbalances towards mainstream health workers  - Too much work for staff  - Successful relationships primarily depend on motivation of individual staff  - Lack of work space  - Inadequate funding | - Role in the development, delivery, and development of the evaluation, participated in the evaluation  - Strong Women Workers work collaboratively with local and visiting health staff and provide cultural education to younger staff | -Decline in low birth wts, improved partnerships, wider social and economic benefits  - Critical conditions for effective and sustainable implementation of the program are often not met  - Program has survived for more than 20 years because of its ivalue to individuals, communities and the Dept of Health |
| Martens, 2002  *Increasing breastfeeding initiation and duration at a community level: an evaluation of Sagkeeng First Nation’s communiyt health nurse and peer counselor programs* | Not stated in bio | CAN  MB  Sagkeeng First Nation  First Nation mothers and babies who resided on- reserve | Mixed methods  Chart audits and client survey  Qual interviews  271 charts audited.  22 interviews (13 peer counsellor clients and 9 non-clients) | -Evaluate the effectiveness of the breastfeeding initiatives  - To increase initiation and duration rates of breastfeeding | - Prenatal education by community health nurse in home or clinic using culturally appropriate resources developed for the community  - Postpartum peer with mothers | - Universal program, all community members eligible  - Culturally appropriate resources developed  - Program was simple  - Changing how questions asked (from how going to feed babies into what they knew about breastfeeding)  - Community Elders viewed breastfeeding as the desirable traditional norm | - Unable to determine coverage of peer counsellor program as clients became non-available, weaned baby, moved away  - Missing chart information about birthweights, parity, weaning dates and breastfeeding duration  - Family members (mothers and grandmothers) more supportive and knowledgeable about bottle feeding than breastfeeding  - Varying levels of social support for breastfeeding | -CHNs decided on long-term strategy for BF promotion in the community, developed the resources, and delivered the program  - Collected evaluation data, participated in interviews, unknown if contributed to design | -Initiation increased from 38% in 1995 to 60% in 1997 with CHN clients half as likely to wean  - 61% were still breastfeeding at 2 months and 56% at 6 months  - Peer counsellor clients reported fewer problems with BF, greater satisfaction, and less favorable bottle-feeding beliefs  - Contrary to quant findings, women reported increased confidence in BF  - No impact on duration rates  - Clients reported greater access to information  increased breastfeeding confidence |
| McCalman et al., 2015  *Empowering families by engaging and relating Murri way: a grounded theory study of the implementation of the Cape York Baby Basket Program* | No  3^rd^ author is affiliated with Apunipima Cape York Health Council | AUS  Cape York Region  Indigenous women  Murri mothers and babies | Constructivist Grounded Theory  Qual interviews and focus groups  N=28  7 women who had received baskets  3 family members  18 healthcare workers (8 Indigenous workers) | -To provide a theoretical framework which explains the process of implementation  - Program aimed to improve the attendance and engagement of Indigenous women at antenatal and postnatal clinics to better maternal health and child health | - Baby Basket Program developed and implemented by the community-controlled health organization - Offers 3 baskets of maternal and baby goods associated health education (primarily by Indigenous health workers) | - Engaging and relating Murri way,  Yarning, holistic wellbeing, extended family included  -Building relationships, trust, respect  -Organizational values and service approaches (family centered, community controlled) to practice  - Responsive to community needs and feedback  - Creating a culturally safe practice –IHWs possess local knowledge of social and cultural dynamics, speak the language  - Home visits  - Resources in plain English and visual images  - Flexible approach  - Link to other services  - Empowering families | - Remoteness of communities influenced program provision (lack of goods, affected family’s ability to make healthy choices, i.e. access to healthy)  - Women leaving community at 36 weeks to birth  - Keeping up with the demand of clients due to high birth rate  - Workers stretched to keep up  - High staff turnover  - Responsibilities of participants, i.e., looking after other children, influenced engagement in program  - For staff, logistics | -Unclear if had role in program development, delivered the program and participated in the evaluation | - Behavior changes such as smoking cessation, healthier diets  - Taking responsibility for health; becoming empowered  - Advocating for community changes  - Workers concerned about effectiveness of shifting behaviour  - Evaluation led to a revised program incorporate the results and extended the home visiting program to from pregnancy to birth and up to child’s 3^rd^ birthday |
| McCalman et al., 2017  *Family-centered interventions by primary healthcare services for Indigenous early childhood well-being in Australia, Canada, New Zealand and the United States: A systematic scoping review* | No  5^th^ author is affiliated with Apunipima Cape York Health Council | AUS,  CAN,  NZ,  US  Indigenous children from conception to age 5. and their families | Systematic Scoping  Review  N=25  Articles | -To outline the current evidence base for family-centered interventions, examine enabling factors, describe the outcomes for Indigenous early childhood well-being  - To inform implementation of Baby One Program by Apunipima Cape York | - Family-centered care that provides support and address wellbeing concerns, often in the family’s home | -Competent and compassionate program providers (non-judgmental, inter-personal skills)  - Cultural competence of Indigenous workers (different languages, family and cultural issues)  - Trusting relationship with client (not dependent on Aboriginal provider)  - Flexible access (in-homes, transport provided)  - Linking across services; collaboration b/n agencies  - Leadership essential to effective partnerships and collaboration  - Culturally supportive care  - Being community- driven  - Incorporating culture, lore, education | - Few impact evaluations completed, only one rated strong quality  - Lack of reporting to the extent to which families are engaged, focused on describing intervention, acceptability, outcomes, feasibility  - Mostly recognize the structural/political determinants, but respond with interventions on behavioral/lifestyle  - Roles of fathers not considered or included  - Lack of economic evaluations | -Reported program deliverers were Indigenous health paraprofessionals, workers, other healthcare professionals, Elders and partnerships with researchers | -For Indigenous children improved nutritional status, emotional, and behavioral and preventive health  - Parents and caregivers reduced depression and substance abuse, improved parenting knowledge, confidence and skills  - Improved service utilization, client satisfaction, cost of delivery  - Evidence suggests family-centered interventions improve Indigenous children’s and parents/caregivers health outcomes, satisfaction with and utilization of healthcare, and community/cultural revitalization |
| Moffitt & Dickinson, 2016  *Creating exclusive breastfeeding knowledge translation tools with First Nations mothers in Northwest Territories, Canada* | Not stated in bio | CAN  NWT  Thcho women in the community  (Dene) women who are pregnant and/or breastfeeding | Quant  Chart Audits  N=198  Qual  Semi-structured interviews  N=9  8 Thcho mothers  1 Elder | - To determine the rate and determinants of exclusive breastfeeding  - To create knowledge translation tools to enhance BF  - To improve prenatal and postnatal care of Indigenous women and children | - An information photobook and video were developed and shared with community, mothers attending community prenatal classes and with the health centre nurses | - Community Advisory Committee (nurse, CHRs, Elder, health services director) in tool development  - Community-specific knowledge translation tools  - Local artist provided artwork of mother and infant  - Video reviewed for cultural appropriateness  - Pulls to breastfeeding included traditional feeding method, spiritual practice, economical option, increased bonding with infant, and identifying it as a healthy practice | - Photobook layout and video script initially developed by researchers from Western lens  - Committee identified sig changes needed in resources as developed from western lens  - Nurses supplemented breastfed babies with formula without explanation or rationale  - Preference of formula, drug & alcohol use, advertising, limited role models  - Medical model for childbirth removes women from community for birth | - Participated in the development and delivery of resources to clients  - No stated evaluation plans | - No evaluation of tools at present  - Maternal care enhanced with a peer support BF group and in-home prenatal outreach by a local nurse  - Multiple factors influence feeding (societal, community, and personal factors)  - Hospital changed to exclusive breastfeeding education  - Program initiated by community |
| Mullany et al., 2012  *The Family Spirit Trial for American Indian Teen Mothers and Their Children: CBPR Rationale, Design, Methods and Baseline Characteristics* | Not stated | US  AI teen mothers and their children from 28 weeks gestation to age 3 | RCT  CBPR  N=322  In 4 communities  Mixed methods assessment  Intervention Group:  Family Spirit plus optimized standard care  Control:  optimized standard care | - To evaluate the impact to reduce health and behavioural risks  - Designed to promote family-based protective factors and reduce behavioral health disparities | - Home visiting (43 session)  - Curriculum administered by AI paraprofessionals  - Parenting education, health promotion, positive relationship with worker | - Family-based approach, preferred by community  - Home visiting to reduce stigma of services, overcome transportation, and other access barriers  - Influence other family members  - Content determined in collaboration with teen parents, community advisory boards  - Highly visual format with culturally relevant illustrations  - Use of story  - Local workers, Native language speakers  - Flexible  - Appropriate caseloads  - Structured supervision for home visitors  - Positive relationships  - Provide referrals and transportation | - Transportation.  - Lack of resources  - Access barriers  - Substance abuse  - Depression and postpartum depression  - Crisis  - Low levels of education | - Program developed and target population of teen mothers determined with tribal stakeholders  - Unclear if FLWs are included in development  - FLWs deliver the program | - Mothers in intervention group had improved parenting knowledge, greater maternal involvement, and fewer depressive symptoms  - Infants had sig fewer externalizing and internalizing behaviours at 1yr of age |
| Munns, 2010  *Yanan Ngurra-ngu Walalja Halls Creek Community Families Programme* | Unable to find bio | AUS  Halls Creek  Offered to antenatal and all parents with children aged 0-3yrs | Case Study  N=25 families | - To develop and implement an Indigenous-led community-based parenting support program  - Aims to foster parent empowerment with peer support and advance community partnerships | -Universal home visiting  - Foster parent well-being, strategies to address child development, general health and nutrition of entire family | - Community workers integral to success  - Collaborative support and work with community partners and agencies  - Whole family approach  - Flexible program  - Tailored to individual, visits can be outside of home  - Male and female staff (visit families together); support men as caregivers  - Pictorial handouts  - Indigenous team driving program  - Workers non-judgmental  - Strengths-based approach  - Cultural safety-  respect, cultural lore as foundation for program  - Holistic view of health and whole community | - Barriers not identified | -Deliver the program, drive the program (take this to mean development, planning, implementation)  - No comment on evaluation design | - Generally, well accepted by community  - Nurses and midwives in the community provided positive feedback re: the program effect and issues managed by workers  - Improving Indigenous families’ engagement with parent support strategies |
| Murphy & Best, 2012  *The Aborigiinal Maternal and Infant Health Service: a decade of achievement in the health of women and babies in NSW* | No bio found | AUS  NSW  Aboriginal women and babies from conception to 8wks of age | Program description | - To describe the development, eval, and expansion of services  - To increase utilization of antenatal and postnatal services by Aboriginal women  -To improve maternity and infant health outcomes | - Midwives and Aboriginal Health Workers provide antenatal and postnatal care in the community; linked with mainstream services  NSW Aboriginal Maternal and Infant Health Strategy (AMIHS): continuity-of-care model | - Community consultations to design for local context and needs.  - Partnerships, collaboration with other services  - Accessible setting  - Flexible service delivery  - Transportation provided  - Holistic health approach  - Empower women and communities  - Peer support of staff  - Home visiting  - Culturally appropriate  - Aboriginal worker on team  - Partnership b/n Aboriginal Health Workers and midwives  - Strong relationships with community and health services  - Retention of staff  - Trust with clients | - Need improvement in health services for Aboriginal families  - Smoking cessation needs to be a priority  - Inclusion of Aboriginal worker in training and support providing unit | - Deliver the program, participated in evaluation  - Unclear if involved in design of program or evaluation | - Health outcomes of Aboriginal women improving  - Clients trusted and supported the program  - Increased antenatal checks (78%vs65%); decreased preterm births (11%vs20%)  - Improved BF rates (initiation 70%vs67%; still BF at 6wks 62%vs59%)  - Aboriginal women trusted and supported the service  - Evaluation highlighted the importance of support and development opportunities for staff |
| Panaretto et al., 2005  *Impact of a collaborative shared antenatal care program for urban Indigenous women: a prospective cohort study* | Not stated in bio  States “interests in Indigenous health & improving health service delivery. For the past 17 years she has been working in Indigenous health”  Held multiple leadership roles in Aboriginal health | AUS  Indigenous women | Prospective cohort study  N=456  Mums and Babies program intervention group  N=84 women, historical control group  N=540 contemporary control group | - Evaluate the impact of the program for Indigenous women in Townsville  - Program aimed to improve antenatal care and perinatal outcomes | - Integrated team approach of antenatal care with multi-disciplinary team that provides daily walk-in clinics, health promotion, screening | - Integrated service delivered in a “safe”, family friendly environment  - Community-based program  - Based on continuity of care  - Cultural safety  - Colocation of services  - Community input to improve service and develop the program  - Community relationships  - Tailored to local context | - Long-term project  - There does not appear to be clearly identified barriers | - Delivered the program  - Not identified if included in program development (“worked with community”), or evaluation design or participation  - Evaluation appears quant data from women in intervention and control groups | - Increased number of antenatal visits  - Improved timeliness of the first visit  - Increased STI screening  - Sig reduction in preterm births 8.7% vs 14.3%  - No sig reduction in low birth wt or perinatal mortality  antenatal outcomes, and perinatal outcomes |
| Panaretto et al., 2007  *Sustainable antenatal care services in an urban Indigenous community: the Townsville experience* | Not stated in bio | AUS  Indigenous  pregnant women and families with children under age 8 | Prospective quality improvement intervention  Intervention (n=781)  Historical control group (n=84) | -Program evaluation | - Has a young family focus, not traditional model of antenatal postnatal care.  - See description Panaretto et all, 2005 | - Community-driven  - Improved access to care  - Continuous evaluation and a quality improvement framework  - Local capacity building  - Staff retention  - Completed both clinical activities and attending to risk factors | - Barriers not explicitly identified or discussed  - Areas for further research include exploring barriers to program attendance for women with >4pregnancies | -See Panaretto et al., 2005 | -Increased antenatal visits  - Sig. reduction in perinatal mortality (14v60 per 1000 births)  - Improved perinatal outcomes  - Gains have been sustained  - Use of program increased from start and maintained over 6yrs (60% of Indigenous pregnant women in Townsville using service) |
| Patten, 2012  *Tobacco cessation intervention during pregnancy among Alaska Native women* | Not identified in bio | USA  Alaska Native pregnant women in Yukon Kuskokwim | CBPR  Descriptive  N=35  Control condition – brief counselling at the first prenatal visit | -To reduce tobacco use among pregnant women | -Counselling by AN counselor, pregnancy and culturally specific self-help materials and quit strategies video | - Based on community identified need  - Personal story as acceptable and effective intervention component  - Local community in educational video  - Positive cultural activities | - Low participation rates suggest program not feasible or acceptable to women  - More work to explore increasing attendance and community beliefs around tobacco use  - Women stated stigma associated with attending program  - More objective information on the risks of tobacco for the baby  - Time constraints | - Counsellor delivered the program  - No identification if involved in program development or evaluation | - Low rates of participation  - Poor tobacco abstinence outcomes  - Need alternative approaches to enhance the reach and improve the efficacy |
| Public Health Agency of Canada,  2012  *Evaluation of the Aboriginal Head Start in Urban and Northern Communities Program at the Public Health Agency of Canada* | No | CAN  Aboriginal preschool children and their families living off-reserve | Program Evaluation  Reviewed literature, key internal documents, case studies, interviews, and focus groups | - Evaluate the relevance and performance of the program  - Program aims to enhance the spiritual, emotional, physical and social well-being of Aboriginal children under 6 and their families | - Centre-based preschool program for 3-5year old running 3-4 half days per week September to June  - Funded from PHAC | - Holistic program (spiritual, emotional, physical, social well-being)  - Aboriginal community organizations design and deliver program  - Community-based approach  - Responsiveness to local needs  - Targets families and communities, not just children  - Strong emphasis on parental involvement  - Culture and language in program design and delivery  - Staff from Aboriginal community  - Local, design ownership and decision making  - Stable staffing  - Transportation  - Low cost  - No standard curriculum | - Need to reach more children  - More collaboration with other federal depts, provinces and territories, stakeholders  - Need review of design, delivery, and governance structure  - Staff turnover  - Increase partnerships  - Some not at full capacity  - Explore contextual factors for success  - Funding levels  - Limited hours of programming  - Less reach to broader community, including Elders  - Limited evaluation  - Regional evaluations not standardized | - Deliver the program  - Participated in evaluation  - Development of program, evaluation not stated | - Program highly regarded by participants  - Positive effect on school readiness, improved language, social, motor, academic skills  - Improved cultural literacy, enhanced exposure to language, culture  - Health promoting behaviours – access to physical activity, nutrition  - Increased access to other health services  - Evidence program sites have created a sense of community for children and families |
| Richer et al., 2018  *Supporting Indigenous families in the Cree territory: lessons from the A Mashkupimatsit Awash (AMA) initiative* | Not stated in bio    States “long experience working in field of Indigenous health” | CAN  QUE | Discussion Paper  Qual  Evaluation  Interviews  n=44  Group discussions  n=2 | - To enhance the health and well-being of pregnant mothers, young children and their families  - Describe the adaptation of the program to an Indigenous context and program implementation challenges | - Inter-disciplinary care and follow up by Indigenous female FSWs | - Local Indigenous workers to ensure programs are culturally safe  - Culturally adapted training  - Ongoing support provided to FSWs  - Family centered  - Responds to families’ unique needs  - Continuity of Care and integration of services  - Community-driven - Aimed at living conditions & healthy, safe child development  - Relationship, trust  - Flexible times, meeting places  - Universal service to avoid stigmatization  - Voluntary program  - High collaboration among teams | - Lack of integration of traditional practices, required Elder support  - Increased support of FSWs needed, felt unqualified.  - FSWs felt challenged to complete visits  - Overcrowded, lack of privacy, dysfunctional homes  - Fear to do visits  - Training too theoretical  - FSWs feeling isolated, not considered equal team members, team conflict  - Burnout and added stress of responsibilities of work and community  - Communication issues with non-Cree | - FSWs deliver the program  - Health care workers were consulted re: program development, participated in evaluation  - No commentary on evaluation design | - Home visiting component did not take place to extent expected  - No specific client outcomes identified from evaluation |
| Schwartz2015  *Promising practices in First Nations and Aboriginal Maternal and child health programs: community perspectives on what works* | Yes  Métis | CAN  B.C.  First Nations women and families | Qual  Interview and group discussions  Health directors, maternal child program coordinators from First Nations communities, health authority, gov’t staff responsible for B.C. maternal child health | - To provide advice and recommendations in BC Tripartite First Nations and Aboriginal Maternal and Child Health Strategy Table of what works, what does not, and areas for improvement | - No specific program described | - Community-based  - Holistic, flexible, culturally grounded approaches and safe  - Community-specific  - Should be universally available  - Woman and family centered approach  - Strengths-based  - Meet families where they are at  - Includes extended family (i.e., fathers)  - High functioning, collaborative teams  - Including Elders, traditional healers  - Consider formal and experiential knowledge  - Trauma informed  - Opportunities for parents to make new friendships  - Community leadership  - Right staff  - Friendliness and hospitality  -Evaluation measures important to the community | - Evaluation that is focused on a list of activities and outputs  - Pathologizing  - Working in silos  - Lack of access to specialists  - Privacy, safety concerns in home visits  - Lack of evaluations  - Lack of flexibility - Lack of coordination  - Funding and service inequities  - Increase investment in programs  - Provide training and support  - Allow communities flexibility  - Collaborate | -Program delivery, participation in evaluation | - Specific outcomes not reported  - Suggested outcomes include births, children in care, breastfeeding, happiness  - Reported what worked, what didn’t |
| Sivak et al., 2008  *A pilot exploration of a family home visiting program for families of Aboriginal and Torres Straight Islander Children* | Not stated in bio | AUS  Aboriginal families with children up to 2 yrs old | Cross-sectional Qualitative design  5 focus groups and 23 interviews  60 families | -To gain the perspectives of Aboriginal clients, staff, mx of what successfully engages and provides service to families of Aboriginal children | - Home visiting service, one visit for each child born in the state, criteria-based additional supports up to 34 visits and age 2  - Visited by a nurse and Indigenous Cultural Consultant  - Provide information, activities, services, and referrals | - Staff characteristics (relational skills, respectful, non-judgmental, open, friendly, genuine)  - Staff seen as more than professionals (often as family or friends)  - Strength-based approach  - Flexibility  - Importance of staff continuity  - Cross-cultural staffing, importance of ICC to families, understanding clients’ context and culture, role in mediating communication, role models for children and community  - Supporting families through contextual challenges such as housing (determinants of health)  - Assisting with transportation  - Whole family approach | - Better communication surrounding program  - Multiple staff and roles can be confusing for participants  - Would like more contact with ICCs  - Close community connections create challenges for ICCs  - Families not eligible for the program, need support  - Fear of child surveillance  - Need male staff and more for fathers, men’s parenting  - Families faced challenges including: Substance use, housing issues, transport, mental health, isolation, family conflict | -FLWs deliver the program, participate in the evaluation, no commentary on role in development or evaluation design | - Participants reported a positive approach to child health and development, empowering, and respectful  - Participants more confident in themselves and their parenting  - Included benefits to parenting older children  - Increased awareness of infant health |
| Smith et al., 2007  *‘Making a difference’: A new care paradigm for pregnant and parenting Aboriginal People* | Not identified in bio | CAN  Aboriginal pregnant women and families | Case Study  Participatory research principles  Interviews and group discussion  N=57  17 comm leaders  25 providers  15 comm members | -Describing community views on safe and responsive care | - Care for pregnant and parenting Aboriginal People | - Understanding the impacts of colonization on experiences and relationships with healthcare  -Strengths-based approach  - Community priorities  - Sense of ownership  - Recognize relevant outcomes to community  - Individuals accessing care reflects program safety, i.e., marginalized  - Accessing care early rather than in crisis  - Empowering approaches to evaluation  - Partnerships with communities, organizations, providers  - Adequate time and resources | - Measures externally imposed, need to be tailored to community.  - Colonizing approaches to evaluation | - Delivery of program  - Participated in evaluation  - No commentary on design of program or evaluation | - Improving individual and collective capacity  - Outcomes reflect long-term turning around of intergenerational impact, but also behavioural, population, biomedical  -Unclear what the program outcome was |
| Smylie et al., 2016  *Understanding the role of Indigenous community participation in Indigenous prenatal and infant-toddler health promotion programs in Canada: A realist review* | Yes  Métis | CAN  Indigenous prenatal and infant-toddler (0-6yrs) | Realist Literature Review  17 articles and 6 program reports describing 20 programs | - To identify programs that demonstrate a positive impact on prenatal or child health outcomes  - Understand the contexts of programs in Canada | Variety of prenatal or child health (0-6yrs) promotion programs and intervention | - Community-based, governance/management of program  - Program integrated with local community infrastructure  - Program content and processes that reflect local knowledge, skills, beliefs, values, and practices, kinship systems  - Builds local capacity  - Stakeholders endorse program  - Promotion of Indigenous ways of knowing, being  - Linked across parental, child, community levels  - Community leadership and participation vital  - Community ownership essential to participation, align programs with local Indigenous knowledge, social systems | - Less likely to have positive outcomes in programs with little community investment and ownership  - Resources developed outside community lack relevance  - Mainstream approaches have unresolved epistemological assumptions when applied | - Local health workers should have led development and evaluation  - Are delivering programs | -Community activation results in program outcomes across health domains and community contexts; higher rates of participation; sustained participation; greater motivation to adapt behaviour  - Investment-ownership is linked to positive program results, magnitude of impact may vary |
| Stout and Harp, 2009  *Aboriginal Maternal and Infant Health in Canada: Review of On-Reserve Programming* | 2^nd^ author  Member of Peter Ballentyne Cree Nation | Canada  First Nations/  Inuit | Review | - To assess Aboriginal maternal and infant programs to determine which populations may be under-served or un-served | - Multiple programs are reviewed (i.e., Aboriginal Head Start On-Reserve, Brighter Futures, COHI, targeted immunization strategy, and Canada Prenatal Nutrition Program) | - Need sustainable funding  - Involvement of Elders and Aboriginal organizations to adapt materials and deliver training  - Culturally relevant resources increase participation in programs  - Projects sufficiently funded  - Aboriginal staff  - Community flexibility to direct resources to specific needs | - Gaps in service remain  **-** Lack of sustainable funding, competitive funding processes  - Staff recruitment, burnout, and retention  - Staff external from community  - Lack of culturally sensitive staff  - Jurisdictional gaps, lack of coordination amongst gov’t, agencies, programs  - Reporting time consuming and laborious  - Gaps in available data  - Limited resources (training, funding, infrastructure) | -Delivering the program, no description outside of delivery | - Outcomes for most projects was the amount of people served  - Communities served are mostly quite high  - Exceptions include CPNP Nunavut (15%) or Children’s Oral Health Initiative (22%) |
| Tomayko et al., 2016  *The Healthy Children, Strong Families intervention promotes improvements in nutrition, activity and body weight in American Indian families with young children* | Not stated in bio  Was stated that the partnering communities had opportunity to review and comment on data and manuscript prior to publication | US  AI adults and children 0-5yrs | Family-based  RCT  CBPR  4 AI communities  Intervention:  n=44  HCSF Toolkit Curriculum delivered by mentor  Control:  n=54  Toolkit delivered by mail monthly | - To assess the efficacy of the program that was designed and delivered using CBPR approaches  - To reduce obesity and unhealthy habits in American Indian preschoolers | - Toolkit with curriculum (12 culturally appropriate lesson plans, activities, and resources) delivered by mentor at monthly home visits | - Community input in program design  - Great acceptance of tool kit  - Child became change agent for family  - Including both adults and children strengthened the program delivery and the efficacy  - Fostered increased family time  - Designed for use in the home environment  - Culturally sensitive program  - Supported by the community  - All communities have secured own funding to continue with projects | - Lack of social support  - Environmental barriers (high cost of healthy foods, lack of time to practice healthy behaviours)  - Sig. program dropout, especially in intervention groups  - Potential burden of scheduling visits, trust issues to allow person in home  - Mentor turnover  - Unable to address other related factors such as stress, poverty, historical trauma, substance use  - Interventions need to be paired with multi-level community-based interventions (schools, worksites), built environment | - Designed toolkits, had input in data analysis.  - Delivered the intervention  - No commentary on evaluation development, does not appear to be participants in evaluation | - No sig. effect of interventions group vs control  - Sig. improvements in both groups exposed to toolkit  - Obese child participants reduced BMI, no change in adult BMI  - Child fruit and vegetable consumption increased, tv watching decreased for children and adults  - Adult self-efficacy for health-related behaviour change and quality of life increased  - Increased family time |
| Turner et al., 2007  *Randomised clinical trial of a group parent education programme for Australian Indigenous families* | Not identified in bio  Talks about working with Indigenous research | AUS  Queensland  Indigenous families | RCT  Repeated measures,  group design Pre/post  intervention with 6 months follow up  4 sites  n=26  intervention  n=25  waitlist control | - To assess the impact and cultural appropriateness of th adapted Triple P (Positive Parenting Program) for Indigenous families  - Program aims to help parents acquire new knowledge and skills | - An 8-session program conducted in groups of 10-12 parent to develop strategies to cope with behaviours and development  - Aims to promote positive, caring relationships between parents and children | -Flexible service delivery; families can withdraw and resume contact  - Respected and valued culture  - Tailored resources; images, language delivery format  - Time for discussion and sharing stories  - Parents liked sharing experiences, felt not alone  - Supported families who had competing demands  - Offer alternative individual sessions  - Culturally acceptable to participants | - Need to engage families when first make contact with program, i.e., low attendance in waitlist group  - Competing demands for families (family crisis, Elder’s business)  - Improve engagement  - More time in group sessions  - Sample size and retention an issue. | - Child health staff and IHWs co-facilitated the parent groups  - Extensive consultations to develop program that appears to include FLWs (as above staff and IHWs)  - Does not identify who or if any inclusion in evaluation design (has lots of questionnaires and surveys) | - Parents reported a sig. decrease in rates of problem child behaviour  - Less reliance on some dysfunctional parenting practices  - Behaviour scores improved  - Maintained effects at 6-month follow-up.  - Program resources, content, process generally positive results  - Some parents access other services based on the positive experience with the program, reported wouldn’t have prior to program participation |
| Walker, 2010  *An evaluation of Ynan Ngurra-ngu Walalja: Halls Creek Community Families Program* | Not stated in bio | AUS  Indigenous families and children | Mixed methods program evaluation  Stories of program participant, staff, and stakeholders  12 families  15 project coordinator and CCWs  22 stakeholders | - Examine how participants believe program has increases self-empowerment, knowledge, and understanding of parenting roles and improved health and well-being | - Home visiting program  - Program previously described in Munns 2010 | - Aboriginal Ways of Working, taking families out bush, interpreters, recognizing family links  - Male CCWs to engage with fathers.  - Relationships of CCWs to family groups  - Holistic approach to health and well-being beyond individual to include extended family and community  - Community-based or community-controlled  - Welcoming safe environment  - Flexible service delivery  - Ongoing training and education  - Program adapts to needs of local community  - Low staff turnover | - Additional roles of CCWs  - Coordinator has little decision-making power  - Lack of awareness and purpose of program in community  - Staff burnout and overextending  - Lack of local resources  -A lack of culturally appropriate services and delivery in other agencies  - Lack of agencies to collaborate with program  - Many issues outside program create negative impacts  - Staff team issues- Depression, substance abuse, domestic violence | - Staff involved as equal partners in evaluation  - CCWs delivered the program | - Enhanced sense of empowerment and control reported by caregivers  - Positive effect for parenting and maternal care  - Increased agency linkage  - Enhancing social networks  - Increasing Aboriginal groups accessing program  - Positive influence on child health outcomes, environmental health, culture and language  - Large number of families participating  - Linking families with other services |
| Whitmore et al., 2018  *Lessons learned and next steps for building knowledge about Tribal maternal, infant, and early childhood home visiting* | Unable to find bio | USA  AI/AN  Families and children prenatal to kindergarten entry | Discussion paper | -Discuss the challenges and opportunities from the Tribal MIECHV program | - Deliver home-visiting services to families and children prenatally to kindergarten for AI/AN communities | - Strength-based approaches, build on assets  - Relationship, trust building between staff, leaders, clients  - Tribal community stakeholder involvement  - Capacity-building  - Alignment with resources and expectations  - Tribal values  - Culturally adapted  - Honor community norms  - Indigenous ways of knowing  - Community voice  - Community knowledge essential  - Acknowledging historical trauma  - Overcoming history of mistrust with research and evaluation  - Community-based evaluation, community inclusion, more meaningful evaluation | -Need a lot of time to build relationships, planning, capacity-building  - Need clear expectations, guidance, support re: data requirements, measures  - Need scientific and culturally rigorous evaluation (tribal evaluation advisory committee)  - Sustainability of funding  - Training, supporting and retaining staff | - Delivered the program  - FLWs included in the development, planning, and evaluation formation | - No specific results or data outlining program outcomes  - Suggest promising program while offering the "lessons learned" |
| Wright et al., 2019  *How Indigenous mothers experience selecting and using early childhood development services to care for their infants* | No  Identified as non-Indigenous in a different article | CAN  ONT  Indigenous mothers of infants less than 2 yrs old | Interpretive Description  Two- Eyed Seeing framework  Interviews  n=19 Indigenous mothers  n=7  PHNs, Family Home Visitors, Indigenous staff | - To explore experiences of Indigenous mothers who select and use ECD services and how to promote access and use | - Non-Indigenous and Indigenous-led ECD programs overviewed | - Comprehensive care, connecting  - Addressing specific and unique needs  - Tailoring teaching  - Providing tangible resources -transportation, groceries, bus pass  - Home visits facilitate access  - Promoting engagement in program – text messaging, incentives  - Indigenous-led  providers demonstrated an awareness of unique contextual factors  - Strengths-based  - Indigenous-led programs better address cultural contexts, i.e., holistic approach  - Prioritized family advocacy  - Understand historical, ongoing trauma  - Long-term relationships | - Text messaging increased work load  - Challenging to build trusting relationships when overworked, short-staffed  - Need better awareness of local Indigenous-led resources  - Need adequate funding to support training for culturally safe and trauma and violence informed care approaches to practice  - Funding and policy to support Indigenous-led organizations  - Strategic partnerships b/n primary, acute and community care services  - Transportation  - Health care providers don’t know about the programs | - Deliver the program  - Not stated other involvement | -Indigenous led programs promote access, built long-term relationships  - Met family needs, cultural and spiritual  - Have Indigenous lived experience, provide culturally relevant programming  - Better outcomes than non-indigenous led programs  - More popular among Indigenous users  - Increased access to services, and enhanced engagement |
| Zarnowiecki et al., 2018  *The Australian Nurse-Family Partnership Program for Aboriginal mothers and babies: Describing client complexity and implications for program delivery* | Unable to find bio | AUS  3 sites  Aboriginal mothers and infants (pregnancy to 2 yrs) | Mixed methods  n=276 clients  Data collection forms  Semi-structured interviews, focus groups, reflexive discussions  n=11  Program staff and key stakeholders | -To describe the complexity of clients and how it affects program delivery and modification  - To improve outcomes for Australian Aboriginal mothers and babies; and interrupt poor health, social, and economic disadvantage | - Home visiting program  - ACWs included for cultural safety, support community engagement, and address SDOH | - Pictorial-based content  - Inclusion of multiparity provides new opportunity for mothers to learn  - Flexibility in conducting visits in alternate setting  - Transiency if the new environment is beneficial to the mother | - Extreme economic and social disadvantage  - Lack of education, low or no income, housing insecurity  - Colonization, effect of historical and ongoing trauma  - Time to develop new resources  - Transiency can impact client engagement  - Home environment, overcrowding  - Client complexity, multiple adversities  - Nurses as case managers, program not delivered as intended  - Additional workload and responsibility on staff  - Large geographical distances | - Nurses deliver the program supported by AHWs  - No commentary on program development  - No discussion on evaluation formation | - Implementation and coverage well accepted by clients  - Retention rates comparable to mainstream program |
